# The basement membrane determines the choice of wound healing mechanism across wound scales in the basal eukaryote *Clytia hemisphaerica*

**DOI:** 10.1091/mbc.E26-02-0094

**Published:** 2026-05-13

**Authors:** Jocelyn E. Malamy, Maxwell Sassaman, Manjula P. Mony

**Affiliations:** ^a^Department of Molecular Genetics and Cell Biology, The University of Chicago, 929 East 57th Street, Chicago, IL 60615; University of Wisconsin, Madison

## Abstract

Epithelial wound healing is an essential process in multicellular organisms, primarily driven by lamellipodia-based crawling, purse-string contraction, and collective cell migration. One or more of these mechanisms participate in healing a wound, yet the choice, sequence, and coordination of these processes are poorly understood. Moreover, different mechanisms dominate in different tissues, organisms, wound types, and wound sizes, further complicating our understanding of how cells select among healing mechanisms. In this study, we analyzed wound healing across wound types and spatial scales in the basal eukaryote *Clytia hemisphaerica* (Clytia) to establish a unified model for mechanism selection within a single organism. We demonstrate that lamellipodial crawling and actomyosin cable contractions are sequential, partially redundant processes involved in healing all wounds. Furthermore, the exposure of the basement membrane acts as a central regulatory cue, orchestrating lamellipodia formation, actomyosin contraction, and collective cell migration responses. Remarkably, we discovered that these same mechanisms operate in healing micro-wounds internal to a single cell. This work fundamentally advances our understanding of how diverse healing mechanisms are coordinated to respond to all types of wounds, while the use of a basal metazoan model expands our knowledge of fundamental strategies for maintaining epithelial integrity.

## Abstract (executive summary)

- Epithelial wound healing is crucial for the survival of multicellular organisms, yet the fundamental question of how cells select the best repair strategies and coordinate these mechanisms in diverse wound types remains unclear.
- Using the medusa of Clytia, we define a decision tree that determines how wound healing mechanisms are orchestrated in all wound types, and identify basement membrane availability as a key regulator in the choice of mechanism.
- This discovery reshapes our understanding of epithelial wound healing. Using a basal eukaryotic model offers new insights into the evolutionary conservation of healing and could inform clinical innovations for tissue repair.

## INTRODUCTION

Sheets of epithelial cells that cover and protect animals from environmental insults emerged early in the evolution of multicellular animals (Tyler, 2003). Mechanisms to repair damaged epithelial sheets likely emerged simultaneously to protect the bodies of multicellular animals from infection and separate internal compartments from the external environment.

Over a century of experimental work has been done to try to understand how epithelial cell sheets recognize that they are wounded and respond to close the gap and restore the integrity of the sheet. Diverse mechanisms have been described for wound closure in different organisms and wound types, but why one mechanism dominates over others in any given context is unknown. In addition, in many cases more than one mechanism appears to be involved in closing a single wound (Begnaud *et al.,* 2016), but how the mechanisms are coordinated to accomplish healing remains an unresolved question. The different models, cell types, and wounding protocols that have been used by researchers have perhaps added to the confusion. For example, healing of small epithelial wounds *in vivo* and *in vitro*, in both embryonic and adult wound healing models, has been ascribed either to contraction of a multicellular actin “purse string,” lamellipodial-based crawling, or some combination of the two depending on the system (Martin and Lewis, 1992; Bement *et al.,* 1993; Danjo and Gipson, 1998; Wood *et al.,* 2002; Anon *et al.,* 2012; Klarlund, 2012; Abreu-Blanco, Verboon, *et al.,* 2012; Fernandez-Gonzalez and Zallen, 2013; Begnaud *et al.,* 2016; Richardson *et al.,* 2016; Rothenberg and Fernandez-Gonzalez, 2019). In larger epithelial wounds and during re-epithelialization in adult vertebrate tissues, epithelial cells move collectively into the wound region to close the gap (Zhao *et al.,* 2003; Vedula *et al.,* 2013; Theveneau and Mayor, 2013; Pastar *et al.,* 2014; Li *et al.,* 2015; Park *et al.,* 2017; Haensel and Dai, 2018; Bornes *et al.,* 2021), but cells migrate either as coherent sheets or as coordinated, independent cells, depending on the cell type (Poujade *et al.,* 2007; Tsai *et al.,* 2018), and the signals that induce collective cell migration may differ in different systems (Matsubayashi *et al.,* 2004; Tanner *et al.,* 2009; Friedl *et al.,* 2014; Li *et al.,* 2015; Enyedi and Niethammer, 2015; Ozawa *et al.,* 2020; Lu and Lu, 2021; Wu and Wong, 2025). Wounds in these studies have been produced in a wide variety of ways, including scratching or stabbing with wire (Bement *et al.,* 1993; Liang *et al.,* 2007), removal of templates or molds in vitro (Poujade *et al.,* 2007; Anon *et al.,* 2012; Klarlund, 2012), surgically creating surface or full thickness wounds in stratified epithelium (e.g., mouse; chick, *ex vivo* cornea; Martin and Lewis, 1992; Danjo and Gipson, 1998; Park *et al.,* 2017), and laser ablating one or more cells (e.g., *Drosophila* embryo, zebrafish, *C. elegans*; Wood *et al.,* 2002; Xu and Chisholm, 2011; Fernandez-Gonzalez and Zallen, 2013; Richardson *et al.,* 2016). These wounding techniques produce wounds of different sizes and shapes and damage tissues in distinct ways, potentially eliciting very different healing processes. Finally, cultured epithelial monolayers, which have been the source of much knowledge about epithelial cell behavior, lack the authentic extracellular matrix and basement membrane (BM) found in *in vivo* models and therefore may behave differently (Jain *et al.,* 2022; Gordon, 2022). Importantly, there is no single system where multiple wound types and sizes have been studied to try to define the rules that govern all healing processes in one organism.

We recently developed the cnidarian *Clytia hemisphaerica* (Clytia) as a model for epithelial wound healing (Kamran *et al.,* 2017; Lee, Watto, *et al.,* 2023; Lee, O'Malley-Krohn, *et al.,* 2023). Advantages of this animal are: 1) there is a monolayer of large epithelial cells (∼50 µm in diameter) on the surface of the transparent Clytia medusa, allowing high resolution imaging of cell behaviors in live animals; 2) the Clytia medusa lacks vasculature, inflammation pathways or migratory fibroblasts, allowing a focus on the direct response of epithelial cells to wounding *in vivo*; and 3) Clytia epithelial cells, extracellular matrix, BM and wound healing processes closely resemble those of more complex organisms, suggesting that this evolutionarily ancient animal can reveal mechanisms for healing that are relevant across the tree of life (Kamran *et al.,* 2017; Lee, Watto, *et al.,* 2023; Lee, O'Malley-Krohn, *et al.,* 2023).

In the present study, we use the epithelial monolayer on the top of the Clytia medusa bell to probe cellular events and actin dynamics during healing of “micro-wounds” that go completely through single cells, “small” wounds that close by stretching of the cells at the wound margin, and “large” tissue gaps that require collective cell migration to heal (Kamran *et al.,* 2017). We characterize micro-wounds in detail for the first time, revealing that lamellipodia can form from membranes internal to a single cell, that disruption of cell junctions is not necessary to induce lamellipodia formation, and that lamellipodia can distinguish between “self” and “non-self” during wound closure. Comparison of actin dynamics and structures during healing in micro-wounds, small, and large wounds confirms that wounds of all types and sizes follow a coherent decision tree to determine the healing mechanism, and that the availability of exposed basement membrane plays a critical role in mechanistic decisions: 1) Actin rapidly localizes to any membrane adjacent to a wound gap; 2) Cell membranes at the wound margin extend lamellipodia into the wound gap as long as there is exposed BM; 3) An actomyosin cable surrounds the wound while lamellipodia extend but does not contract; 4) The continued presence of lamellipodia and exposed BM after maximal lamellipodia extension triggers collective cell migration to close large gaps; and 5) When lamellipodia meet across the wound gap, or cannot extend because the BM is damaged/occluded, the actomyosin cable is triggered to contract, drawing wound marginal cells together and extruding debris. This integrated model for healing of wounds at all scales in Clytia highlights the ubiquitous and overlapping roles of lamellipodia and actomyosin purse strings in wound closure and demonstrates that the availability of the BM plays a critical role in orchestrating the essential events in wound healing.

## RESULTS

### Micro-wounds between or within single cells rapidly induce lamellipodia formation

**FIGURE 1:**
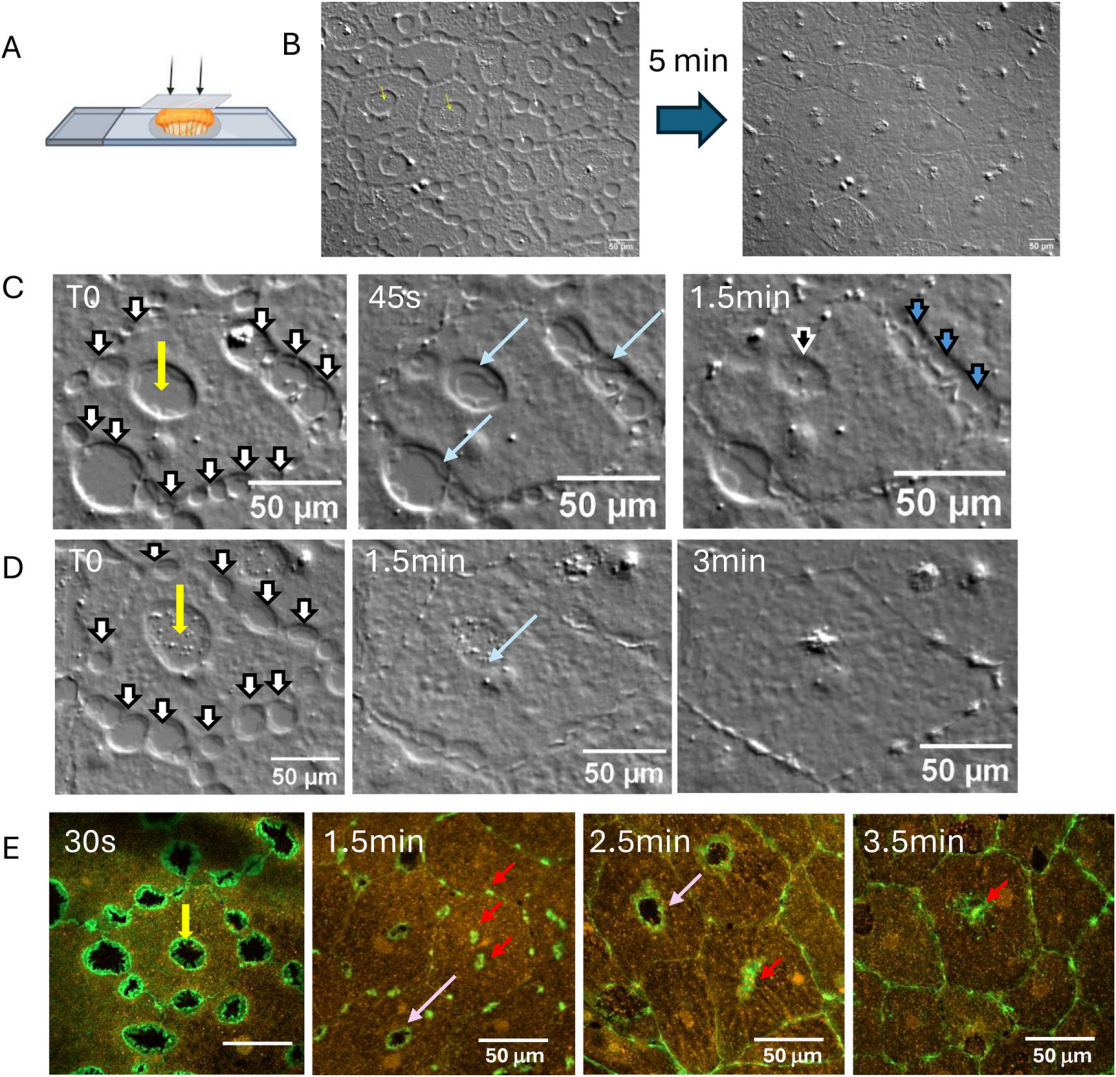
Micro-wound healing in the exumbrella of Clytia medusa. A) Method for creating micro-wounds. B) DIC images of a field of epithelial cells immediately after wounding and 5 min later. C and D) DIC images of micro-wound healing time-course. T0 images are taken 15–30 s after wounding. Left panels: Wounds within (yellow arrows) and between (white arrows) epithelial cells. Middle panels: Lamellipodia form in both wound types (blue arrows). C) No debris is apparent. Lamellipodia in wounds within the cell fuse (right panel, black arrowhead) while those between cells stay separate and re-create the cell margin (right panel, blue arrowheads). D) Debris is present in the wound gap within the cell (left panel, yellow arrow). E) Confocal images of phalloidin-stained actin (green) and Cytoliner-stained membranes (red) in fixed animals after micro-wounding. Lamellipodia are apparent at 30 s (left). By 1.5 and 2.5 min most micro-wounds have closed by lamellipodial extension and actin ring contraction (red arrows). Where there was debris in the wound gap within the cell, actin rings are apparent for an additional 1–2 min (pink arrows) before closure. At the final stages of healing, actin puncta are often seen in a “firework” pattern around the closed wound (3.5 min), but the significance of this is unknown. Scale bar = 50 µm. Images are representative of over 100 micro-wounds in 5 separate experiments. s=seconds

To create micro-wounds, we place a Clytia medusa on a depression slide, bell side up, and press gently with a cover slip (Figure 1A). This creates micro-wounds that pass completely through (Figure 1, B–D, yellow arrows; area = ∼500–2500 µm^2^) and between (Figure 1, B–D, white arrows; area = ∼100–900 µm^2^) cells. (Note that the micro-wounds within cells described here pass all the way through the cell and expose the BM that underlies the epithelial monolayer, unlike well-studied single-cell wounds (i.e., in oocytes) that only create a hole in the plasma membrane.) While the forces responsible for these wounds were not defined, wounds likely result from the stretching of the epithelial sheet, compression of the cells, and/or rebounding of the decompressed mesoglea (“jelly”) after the initial pressure (Casares *et al.,* 2015). Micro-wounds heal within 3–5 min (Figure 1B). Micro-wounds within cells often contain cell debris (Figure 1D, T0, yellow arrow), while other micro-wounds within cells (Figure 1C, T0, yellow arrow) and those between cells (Figure 1, C and D, T0, white arrows) are free of debris. For wounds between cells, adjacent cells are pulled apart but remain attached at regular intervals, creating gaps where regions of adjacent cell membranes are separated to reveal the BM (Figure 1, C and D T0, white arrows).

Lamellipodia-like actin-rich membrane protrusions appear at the margins of micro-wounds both within and between cells within 30 s of wounding and extend into the wound area (Figure 1, C and D, light blue arrows; Figure 1E, 30 s). Between cells, this represents a miniature version of the lamellipodia formation that occurs at cell fronts in larger wounds, and demonstrates that lamellipodial protrusions can be restricted to the subsection of the cell membrane that borders a wound gap. The appearance of lamellipodia in wounds that lie partially or completely within a single cell (Figure 1, C–E, yellow arrows) is more surprising. A likely explanation is that the plasma membrane seals spontaneously around the area of damage, creating a doughnut-shaped cell and a new cell “front” at the wound margin that can generate lamellipodia. This observation indicates that wound-induced membrane protrusions in Clytia are not specific to membranes located at cell:cell boundaries, and loss of cell:cell junctions is therefore not required to stimulate lamellipodia formation.

### Lamellipodia extension and actin ring contraction act together to close micro-wounds and extrude cell debris

We used time-lapse microscopy of live animals (Figure 1, C and D; Supplemental Videos S1 and S2) and phalloidin staining of fixed tissues at multiple time points (Figure 1E) to analyze micro-wound closure. (Note that DIC images follow the closure of specific wounds over time, while fluorescent images show wounds from different animals fixed at the indicated timepoints.) We found that in micro-wounds lacking debris, lamellipodia contact each other in less than 3 min and retract, followed by a rapid contraction of the tissue (Figure 1, C and E; Supplemental Figure S1; Supplemental Videos S1 and S2). Contractions, seen most clearly in time-lapse videos, appear as actin-rich knots of tissue by 1-2 min post-wounding (Figure 1E, red arrows), indicating the contraction of an actin ring. For wounds where there is considerable debris, healing is slower, and the actin ring can be clearly seen after the lamellipodia have retracted (Figure 1E, pink arrows; Supplemental Figure S1). After contraction, the tissue relaxes and appears healed.

Interestingly, for micro-wounds within cells, lamellipodia distinguish “self” protrusions originating from membranes within the same cell from “non-self” lamellipodia that originate from a different cell. “Self” lamellipodia fuse (Figure 1C, 1.5min, black arrowhead), while lamellipodia from adjacent cells remain distinct when they meet (Figure 1C, 1.5min, blue arrowheads; Supplemental Video S3). This suggests that, in Clytia, the newly formed cell “fronts” within a cell are distinct from membranes at the cell periphery, allowing cells to avoid the catastrophic mistake of forming junctions interior to a single cell.

Contraction extrudes debris and excess material from the wound site in all cases. This is particularly obvious for wounds within cells, where cell debris is common (Supplemental Videos S3 and S4), and suggests that the elimination of debris is an important function of actin ring contraction.

### Actin polymerization is not necessary for actin localization to the micro-wound margin

**FIGURE 2:**
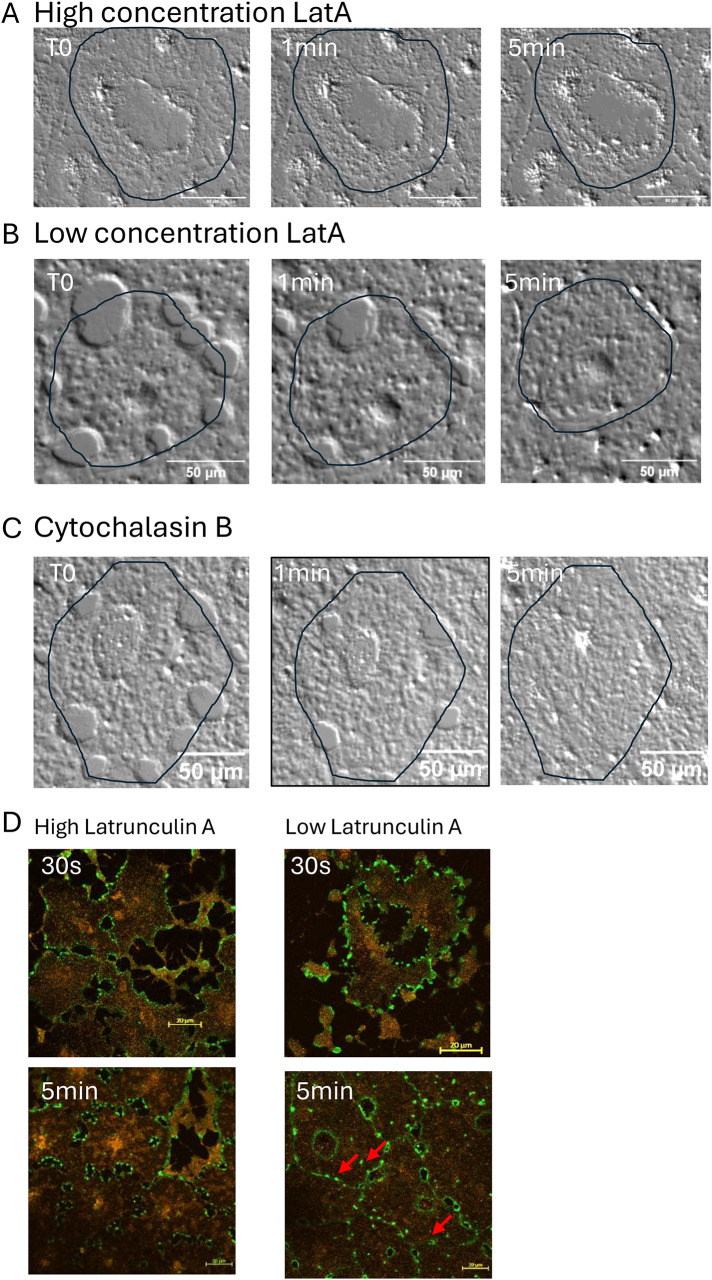
Effect of inhibitors of actin polymerization on micro-wound healing. A–C) Animals were treated for 20 min with 24 nM (high concentration) Latrunculin A (LatA) (A), 14 nM (low concentration) LatA (B), or 1 mM cytochalasin B (C) before micro-wounding. DIC images of one representative cell are shown over time for each treatment. The black line identifies the outline of the cell. In all treatments, membrane protrusions are very small or nonexistent. Micro-wounds within and between cells heal with low concentration LatA and cytochalasin B, but not with the high concentration LatA treatment. D) Confocal images of phalloidin-stained actin (green) and Cytoliner-stained membranes (red) in animals fixed at various times after micro-wounding. No lamellipodia are seen. Actin localizes to the periphery of wounds in a punctate pattern with a high concentration of LatA (left panels), but no actin rings form, and wounds are unhealed at 5 min. In contrast, actin rings form with low concentration LatA (right panels) and most wounds close (red arrows), although actin rings persist in some wounds at 5 min, where there is debris. Scale bar = 50 µm. Images are representative of over 100 micro-wounds in three separate experiments with three to five animals in each experiment.

Accumulation of actin at the cell membrane is a necessary precursor to actin branching to form lamellipodia and actin bundling to form rings. In the presence of high concentrations of latrunculin A (LatA) (24nM), which sequesters G-actin and thereby inhibits de novo actin polymerization, neither lamellipodia nor actin rings form, as expected, and there is no micro-wound closure (Figure 2, A and D; Supplemental Video S5). Actin nevertheless accumulates in a punctate pattern at the wound margin (Figure 2D), showing that this initial localization of actin is not dependent on actin polymerization. This suggests that actin rings in micro-wounds are initially formed from the recruitment of pre-existing actin filaments.

### Lamellipodia extension and actin ring contraction are separable and partially redundant mechanisms of closing micro-wounds

**FIGURE 3:**
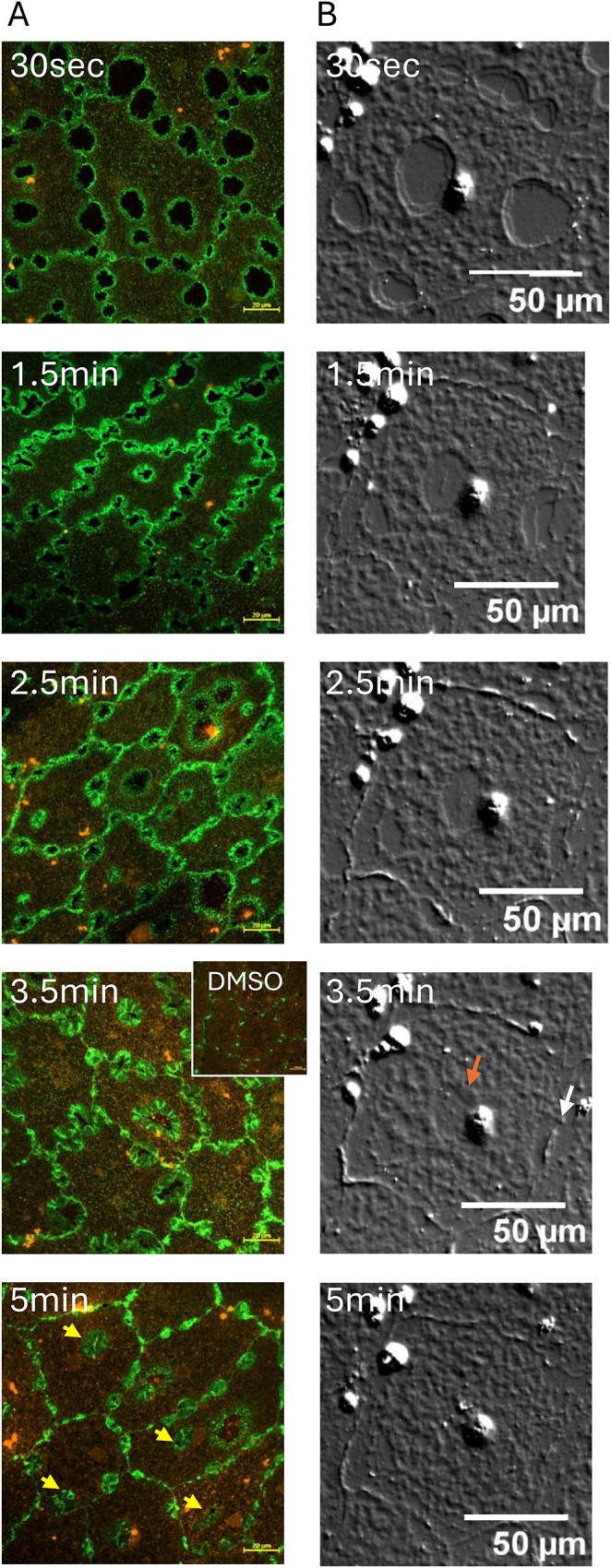
Effect of non-muscle myosin inhibitor on micro-wound healing. Animals were treated with blebbistatin for 20 min before micro-wounding. A) Confocal images of phalloidin-stained actin (green) and Cytoliner-stained membranes (red) in animals fixed at various times after micro-wounding. Lamellipodia are apparent by 30 s post-wounding and are evident both between and within cells at time points where they have disappeared during wound contraction in control (DMSO-treated) animals (compare to inset at 3.5 min). No actin rings form, and no tissue contraction is apparent. In all wounds between cells and most wounds within cells (arrows, 5 min), lamellipodia completely cover the wound gap by 5 min, although some wounds within cells close more slowly. Small actin puncta are sometimes seen surrounding closing wounds, but the significance of this is not known. Scale bar = 20µm. B) DIC images of micro-wound healing in a representative cell reveal lamellipodia but no apparent contraction. Note that lamellipodia within cells fuse to close intracellular wounds (orange arrow) while lamellipodia between cells reconstruct the cell margins (white arrow). Scale bar = 50 µm. Images are representative of over 100 micro-wounds in three separate experiments with three to five animals in each experiment.

To probe the interactions between lamellipodia formation and actin-based contraction in micro-wound closure, we used pharmacological inhibitors. In the presence of the non-muscle myosin inhibitor blebbistatin, lamellipodia formation is robust. Indeed, large lamellipodia, often overlapping, are observed in wound gaps at time points when wounds are completely closed in untreated animals (Figure 3A; compare 1.5 min and later timepoints to Figure 1). However, wounds in blebbistatin-treated animals do not contract, indicating that contraction relies on an actomyosin ring (Figure 3B; Supplemental Video S6). Blebbistatin, therefore, allows the roles of lamellipodia and actomyosin ring contraction in wound closure to be assessed separately. Despite the lack of contraction, micro-wounds in blebbistatin-treated tissues close through advancement and fusion (within a cell) or adherence (between cells) of lamellipodia to each other (Figure 3; Supplemental Video S6). Occasionally, punctate actin is seen surrounding wounds with debris at time points when other wound gaps appear to be completely covered with lamellipodia (Figure 3A, 5 min); this may reflect the difficulty in healing such wounds without contraction to extrude debris.

In the converse experiment, we assessed the role of the actomyosin ring in the absence of lamellipodia. Lamellipodia formation and actomyosin ring formation are differentially sensitive to actin polymerization inhibitors; after treatment with lower levels of LatA (14nM) or with 1 µM cytochalasin B, lamellipodia are almost eliminated, but functional actomyosin rings still form (Figure 2, B–D; Supplemental Figure S2). Interestingly, micro-wounds heal completely in the absence of lamellipodia when treated with these inhibitors (Figure 2, B–D red arrow; Supplemental Videos S7 and S8). This shows that actomyosin ring formation and contraction, and the resulting micro-wound closure, are independent of lamellipodia formation.

Together, these observations show that extension of lamellipodia and actomyosin contraction are partially redundant, separable mechanisms of healing in micro-wounds. Lamellipodia extension fills in the wound gap and is rapidly followed by actomyosin-dependent tissue contraction that pulls wound edges close together and extrudes debris. The existence of two redundant mechanisms likely adds robustness to the wound healing process.

### Small wounds close through sequential lamellipodia extension and contraction of an adherens junction-dependent multicellular actin cable

Having found that micro-wounds heal by sequential and partially redundant mechanisms of lamellipodia extension and actomyosin contraction, we asked whether this healing strategy also applies to wounds at other spatial scales. We previously characterized “small” intercellular wounds, defined as wound gaps that are 1–3 cell widths in diameter, in great detail (Kamran *et al.,* 2017; Lee, Watto, *et al.,* 2023; Lee, O'Malley-Krohn, *et al.,* 2023). Small wounds are made by scratching the top of the medusa bell or by applying gentle pressure to the top of the animal, as for the micro-wounds. Either approach causes the monolayer to rip and the tissue edges to retract from each other due to tension of the sheet, exposing the BM in wound gaps but causing very little cell death.

**FIGURE 4:**
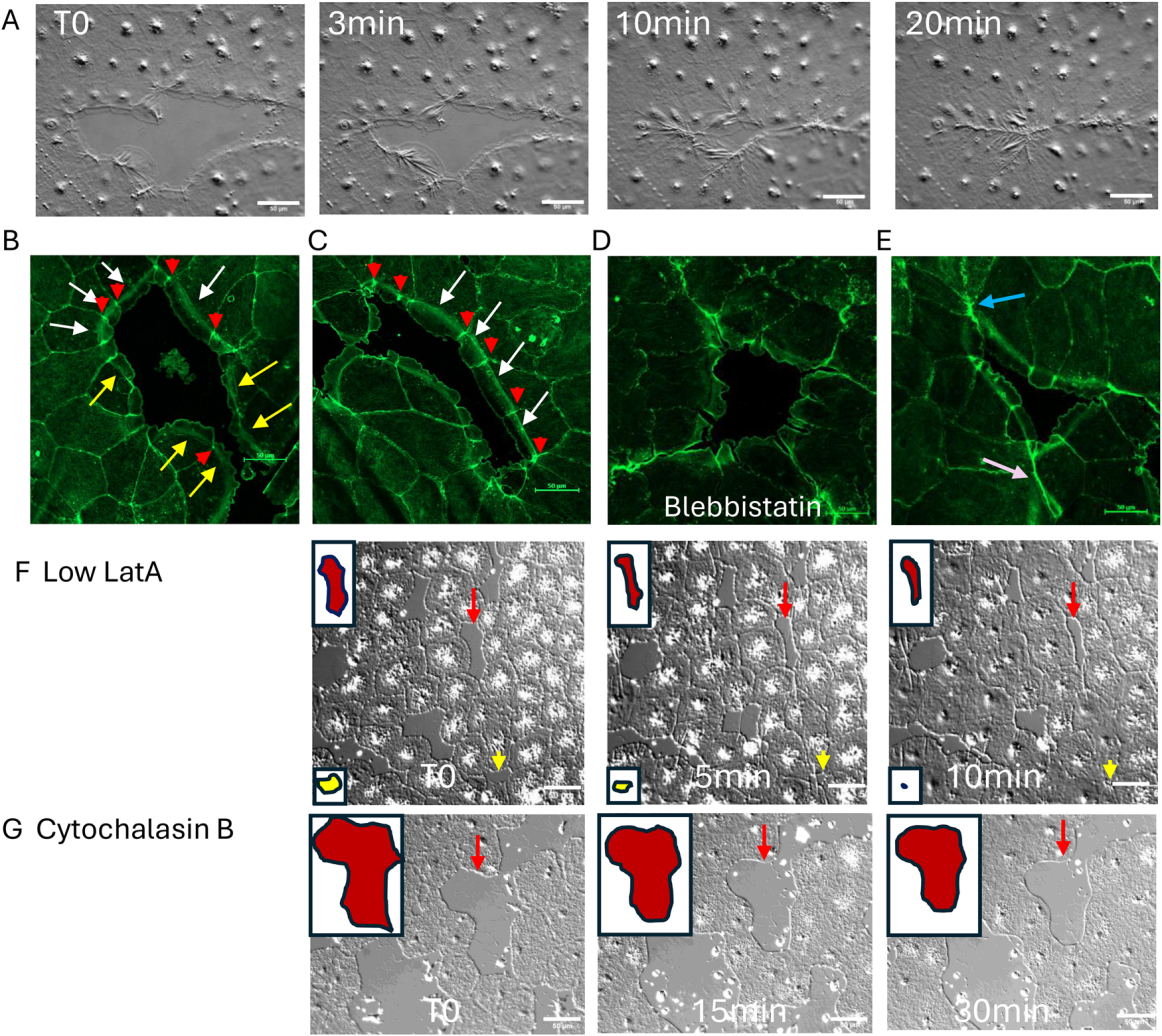
Small wounds close with sequential lamellipodial crawling and actin cable contraction. A) DIC time-lapse of a representative small wound. Lamellipodia extend over exposed BM and, when the BM is covered, the tissue undergoes a contraction. B–E) Confocal images of phalloidin-stained actin in fixed animals at 20 min post-wounding. B and C) Actin is evident at the front of the lamellipodia and at distinct points at the lateral sides of extending lamellipodia (red arrows), revealing nascent AJs. In cells where AJs are just beginning to form, actin within the lamellipodia is diffuse (yellow arrows). In contrast, where the AJs are well developed, cables of actin are seen extending between AJs, forming a multicellular cable (white arrows). D) AJs and actin cables do not form in blebbistatin-treated animals. E) As lamellipodia meet across the wound gap, the actin cable and lamellipodial actin appear to combine (pink arrow) and contract (blue arrow). F and G) Animals were treated for 20 min with 14 nM LatA (F) or 1 µM Cytochalasin B (G) before wounding. No lamellipodia form. Edges of most small wounds contract but are unable to fully close (red arrows). As before, micro-wounds close with contraction alone (yellow arrows). Insets: Shapes of the small (red) and micro (yellow) wounds indicated by arrows are presented to highlight wound contraction. Scale bar = 50 µm. Images of wound healing are representative of 25 small wounds in separate experiments, while images from inhibitor studies represent two separate experiments with three to five animals in each experiment.

In small wounds in Clytia, lamellipodia form from the cells at the wound margin (marginal cells) within minutes and extend across the exposed BM, pulling the cell bodies forward (Figure 4A; Supplemental Video S9) as previously described (Kamran *et al.,* 2017). As previously shown, a non-muscle myosin-dependent contraction of the tissue occurs at the end of the healing process (Figure 4A; Supplemental Video S9; Kamran *et al.,* 2017). To gain a more detailed understanding of the contraction stage of small wound closure and how the lamellipodia and contraction mechanisms interact with each other, we imaged actin at multiple times after wounding. Actin accumulation is first seen throughout the lamellipodia or loosely associated with the lamella (lamellipodia base; Figure 4B, yellow arrows). As wound healing progresses, actin foci become apparent between adjacent advancing lamellipodia, indicating the formation of adherens junctions (AJs) at the lateral sides of these protrusions (Figure 4, B and C, red arrowheads). Actin cables also become evident, extending between the new AJs (Figure 4, B and C, white arrows). Assembly at AJs is a definitive characteristic for multicellular actin cables, or “purse strings,” in a wide variety of systems (Anon *et al.,* 2012; Wood *et al.,* 2002; Danjo and Gipson, 1998). However, the assembly of these cables at the sides of advancing lamellipodia as they form junctions with each other has not been described previously in wound healing. Non-muscle myosin is essential for AJ maturation (Ivanov *et al.,* 2022; Vicente-Manzanares *et al.,* 2009), and indeed, no actin foci were seen between advancing lamellipodia in the presence of the myosin inhibitor blebbistatin, nor were actin cables formed (Figure 4D). This suggests that, as expected, AJs are essential for the formation of multicellular actin cables in Clytia.

The formation of actin cables between the newly formed AJs of adjacent lamellipodia effectively creates a multicellular actin cable surrounding a wound of any shape or size (Figure 4, B and C). Importantly, the actin cable co-exists with the advancing lamellipodia, although little contraction is evident while lamellipodia are present (Figure 4, B and C). In contrast, once lamellipodia extend across the exposed BM and contact each other, the actin in the lamellipodia appears to merge with the actin cable (Figure 4E, pink arrow) and contraction is initiated (Figure 4, A and E, blue arrow; Supplemental Video S9). The triggering of actin cable contraction only after lamellipodia cover the exposed BM identifies an unappreciated regulatory step in the coordination of the two mechanisms. The observation of sequential lamellipodia extension and actin cable contraction in small wound closure reveals a healing strategy that is remarkably similar to that seen in micro-wounds.

We used inhibitors to further probe similarities between small wound and micro-wound healing. To separate lamellipodia extension and actomyosin cable contraction mechanisms, we again used a low concentration of LatA or cytochalasin B to inhibit the formation of lamellipodia but permit actomyosin cable contraction. As shown before, micro-wounds can close by contraction alone (Figure 4, F, yellow arrows). However, although most small wounds show contraction (as evidenced by the decreasing area of the wound, the rounding of the wound shape, and the pinching of the surrounding cells), they do not close even with long observation (Figure 4, F and G; red arrows; Supplemental Videos S10 and S11). We previously showed that with blebbistatin treatment, which blocks both actin cable formation and actomyosin contraction, lamellipodia extend across the wounds but the wounds do not heal (Kamran *et al.*, 2017). Together, these results indicate that lamellipodia extension and actomyosin-based contraction are separable mechanisms of wound closure in small wounds, as in micro-wounds. However, healing of small wounds requires the activity of both mechanisms.

### Retraction of lamellipodia when the BM is unavailable triggers actomyosin contraction in all wounds

**FIGURE 5:**
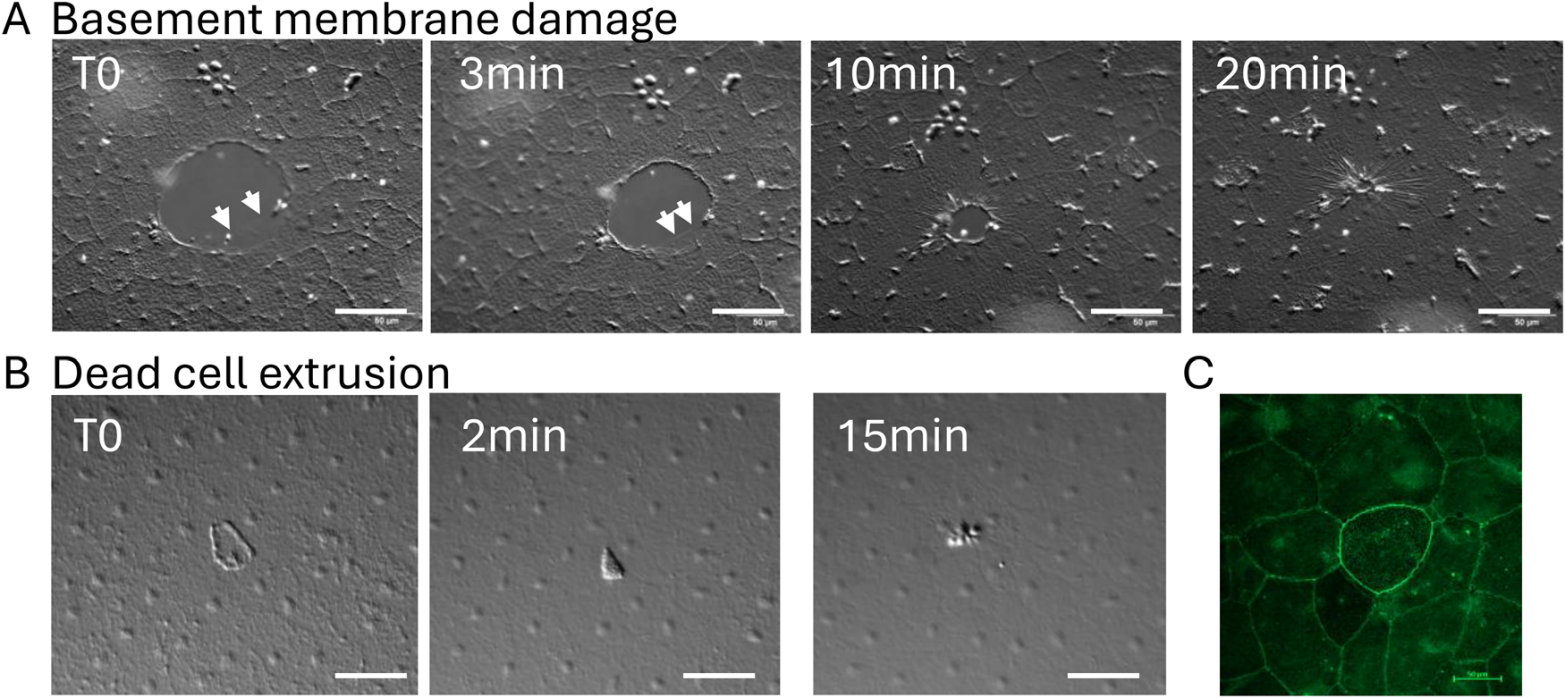
Actomyosin cable contractions close wounds where lamellipodia cannot extend on the BM. A) Time-lapse of actomyosin cable contraction closing a wound where the BM is damaged. The edge of the damaged BM is marked with white arrows at T0 and 3 min. Note the absence of lamellipodia in the area without a BM. B) Time-lapse of actomyosin cable contraction around a dead cell and extrusion of the damaged tissue. C) Confocal image of phalloidin-stained actin reveals the presence of an actomyosin cable around a dead cell. Scale bar = 50 µm. Images are representative of healing in 10 separate experiments.

Multicellular actomyosin purse strings have been extensively described in the closure of small wounds (Anon *et al.,* 2012; Klarlund, 2012; Abreu-Blanco, Verboon, *et al.,* 2012; Bement *et al.,* 1993; Wood *et al.,* 2002; Martin and Lewis, 1992; Fernandez-Gonzalez and Zallen, 2013; Richardson *et al.,* 2016; Rothenberg and Fernandez-Gonzalez, 2019). The “purse string” nomenclature reflects their typical description encircling and closing a circular gap, reminiscent of the drawstring at the top of a purse. In Clytia, we previously reported that lamellipodia rapidly retract when they encounter BM damage in a wound gap, and the wound subsequently closes through a purse string mechanism (Kamran *et al.,* 2017; Figure 5A; Supplemental Video S12). A similar response is seen when a dead cell occludes the BM; in this case, an actomyosin cable surrounds the dead cell and contraction extrudes the damaged tissue (Figure 5, B and C; Supplemental Video S13). Notably, the contraction of the multicellular purse-string in these examples is essentially identical to actomyosin cable contraction during the final healing stage in small wounds (i.e., Figure 4A) except that contraction around a damaged BM area or dead cell typically appears circular, while contraction in wounds already closed by lamellipodia appears linear (Figure 4A vs Figure 5).  Indeed, actomyosin cables are evident in all small wounds, even in the absence of BM damage or dead cells, as shown above (Figure 4). Collectively, these findings indicate that multicellular actomyosin purse strings are not exclusive to circular wounds or those with BM damage or occlusion; rather, they are integral to the healing process in all small wounds in Clytia.

If a multicellular actin cable is a common feature in the healing of all small wounds, there should be a consistent mechanism to trigger its contraction. In small wounds where there is an intact BM, contraction is initiated when lamellipodia meet (Figure 4). However, in cases where the BM is damaged or a dead cell is present, actomyosin cable contraction occurs without lamellipodia formation or contact (Figure 5), indicating that lamellipodial contact is not the trigger.  In contrast, in all the small wounds studied, contraction occurs when lamellipodia are unable to extend—either because the BM is completely covered by lamellipodia or because the BM is damaged or occluded by cell debris. Lamellipodia are transient structures, and without access to the BM, they rapidly retract (Figure 4; Figure 5A). Therefore, we propose that the absence of exposed BM prevents stable lamellipodia formation and acts as a trigger for actomyosin cable contraction. (This hypothesis is also consistent with the contraction observed when lamellipodia formation is prevented by low LatA or cytochalasin B treatment; Figure 4, F and G; Supplemental Video S10 and S11). This is an effective wound healing strategy, as the lack of stable lamellipodia serves as a signal either that the wound gap is closed (with BM fully covered by lamellipodia) or that an obstacle has been encountered by the lamellipodia and must be eliminated by actomyosin-mediated extrusion.

### Lamellipodial extension in large wounds triggers collective cell migration and prevents actomyosin cable contraction

**FIGURE 6:**
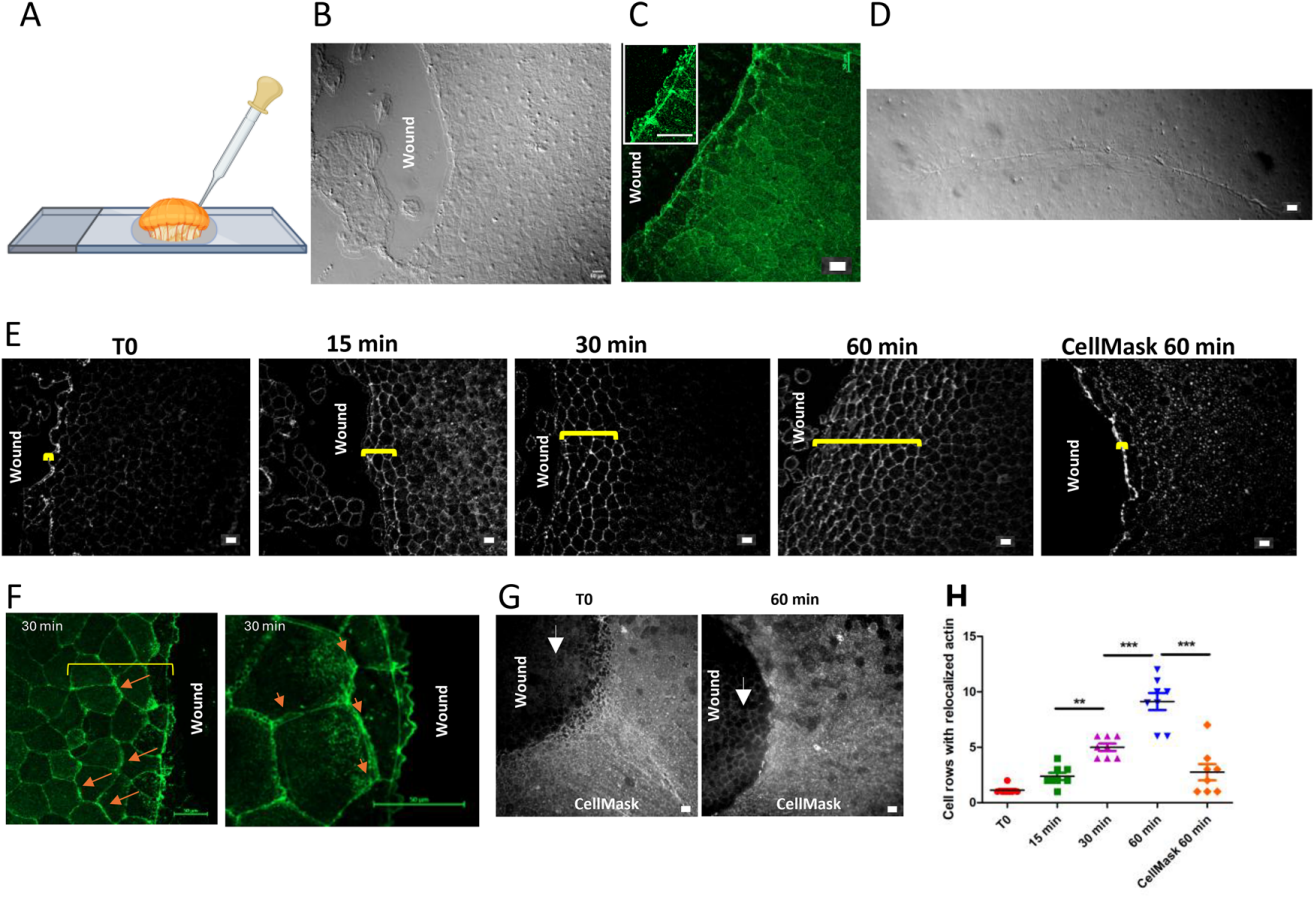
Large wounds induce collective cell migration and progressive actin relocalization in submarginal cells. A) Method for creating large wounds. B) DIC image of a representative large wound. C) Confocal image of phalloidin-stained actin (green) in a representative large wound. Note AJs and actin cable along the wound edge in the image and magnified inset. D) DIC image showing contraction at the final stages of healing of a large wound. E) Fluorescence microscopy images of phalloidin-stained actin in large wounds after background subtraction (raw images in Supplemental Figure S3). The yellow bracket indicates the rows of cells in which actin has relocalized to the cell edges at each time point. F) Confocal images showing actin protrusions at the front edges of submarginal cells. Bracket indicates the rows of cells where actin has relocalized to the cell edge. Red arrows indicate submarginal membrane protrusions. G) Wounds were made by exposing Cellmask-stained cells to light (refer to *Materials and Methods* section). Dead cells stained with Cellmask can be seen adhering to the BM adjacent to the wound (white arrows) immediately after wounding (T0) and 60 min later. Images are representative of eight experiments. (A–G) Scale bar = 50 µm. H) Quantification of rows of cells with actin relocalized to cell edges over time. Eight wounds in six different animals were measured at each time point. Three measurements were taken per wound image and averaged. Each data point represents the average number of cell rows with relocalized actin on one image, including marginal and submarginal cells. Bars represented mean ± SEM of eight images. Statistical differences were tested using Tukey's multiple comparisons test (one-way ANOVA), **P < 0.005; ***P < 0.0001. While actin relocalization was sometimes seen in submarginal cells at 15 min, the difference from T0 was not statistically significant.

To further identify consistent patterns in the choice of healing mechanism in different wound types, we created large wounds by gentle suction on the epithelial sheet with a Pasteur pipette. Again, this treatment causes the sheet to tear and the tissues to retract due to tension, but does not result in many damaged or dead cells (Figure 6, A and B).

Lamellipodia and multicellular actin cables form and co-exist in marginal cells in large wounds, as seen in small wounds (Figure 6C). Lamellipodia have access to the exposed BM in the wound gap and are therefore stable, yet lamellipodia extension is not sufficient to bridge the large wound gap. Consistent with our model that lamellipodia must retract due to a lack of access to the BM to trigger actomyosin cable contraction, little to no contraction is seen in these unhealed large wounds. Instead, marginal and submarginal cells begin to migrate collectively across the wound gap (Kamran *et al.,*, 2017; Supplemental Video S14). Once lamellipodia of the advancing marginal cells meet across the wound gap, collective cell migration ceases and wound healing proceeds as for small wounds, including the contraction of an actomyosin cable after lamellipodia meet (Figure 6D; Supplemental Video S15).

### Mechanical signaling from marginal to submarginal cells triggers changes in actin localization

To better understand how collective cell migration is induced in large wounds, we imaged actin in marginal and submarginal cells. Immediately after wounding, actin localizes to the cell edge adjacent to the wound in marginal cells, as seen in small wounds and micro-wounds, while actin localization in submarginal cells is unchanged compared to unwounded tissues (Figure 6E, T0). However, after 15 min, actin also localizes to the fronts of the submarginal cells immediately behind the marginal cells. Closer examination of the submarginal cells shows that the actin relocalization is accompanied by membrane protrusions along the edge of the cell facing the wound gap (Figure 6F, red arrows). While these protrusions resemble cryptic lamellipodia, we are unable to definitively demonstrate that they extend under the cell in front of them. The induction of actin relocalization and membrane protrusions progresses across the epithelial tissue, spreading ∼5 rows from the wound margin by 30 min and ∼10 rows by 60 min (Figure 6, E and H).

Flow of information from marginal to submarginal cells to induce actin relocalization could involve a diffusible signal, perhaps derived from damaged cells, or a mechanical signal due to cells pulling on the cells to their rear as they migrate. To distinguish between these possibilities, we took advantage of our observation that Clytia epithelial cells are extremely sensitive to phytotoxic dyes. Clytia cells are first stained with the vital stain Cellmask, and then a region of the epithelium is illuminated with green light under a 20X lens for 2 min. The resulting patch of dead cells is approximately the same size as the large wounds previously examined; however, the BM is completely covered with adherent dead cells (Figure 6G, white arrows). This massive cell death would be expected to release any cellular contents that might act as diffusible wound signals. However, induction of actin accumulation and membrane protrusions in submarginal cells is greatly reduced (Figure 6E, right panel; Figure 6H), suggesting that the flow of information from the marginal to submarginal cells is likely not driven by a damage-induced diffusible signal, although we cannot rule out the possibility that wounded cells produce different diffusible signals than cells killed with phytotoxic dye. In contrast, the dead cells that occlude the BM in the Cellmask wounds effectively block stable lamellipodia formation and stretching of the marginal cell into the wound gap. Therefore, we propose that submarginal cells are activated by a mechanical signal, with each row of cells pulling on the row behind it to ultimately create the observed wave of actin relocalization and protrusive activity.

## DISCUSSION

### A coherent decision tree determines the healing mechanisms for epithelial wounds across scales in Clytia

**FIGURE 7:**
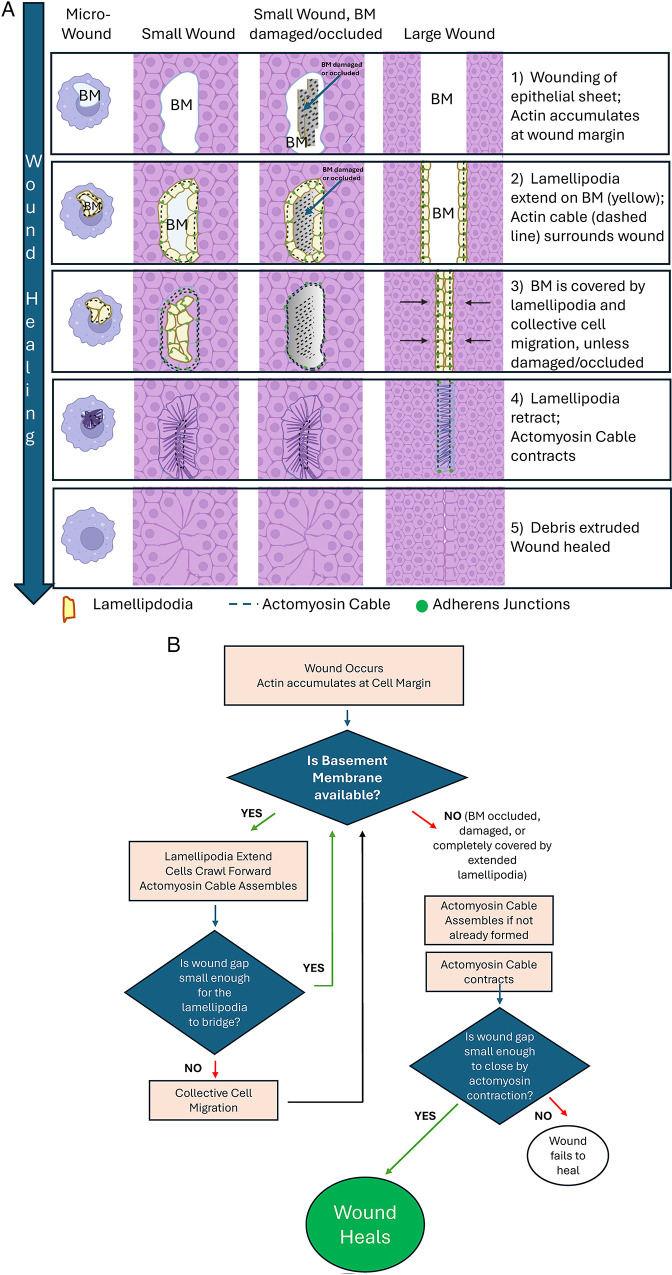
A coherent model and decision tree for the selection of healing mechanisms across all wound types and scales, and over the course of the healing process. A) Schematic representation of the selection of healing mechanisms in distinct wound types. BM = exposed basement membrane; B) A decision tree depicting the central role of BM availability in coordinating wound healing mechanisms during wound closure.

Our observations lead to a model and decision tree that, together, explain the choice of wound healing mechanism for all wound types and spatial scales in Clytia, with the availability of the BM as a key determinant (Figure 7A). First, actin accumulates at the cell membrane(s) adjacent to the wound gap (Figure 7A-1). Lamellipodia form, and an actomyosin cable/ring is assembled at the base of the lamellipodia. The actomyosin cable surrounds the wound but does not contract (Figure 7A-2). Lamellipodia extend until all the exposed BM is covered. In large wounds where the gap is too large to be covered by lamellipodia extension, mechanical signals move from the wound margin to submarginal cells and collective cell migration is activated to cover the BM (Figure 7A-3). When no exposed BM is available for further lamellipodia extension, this signals that the lamellipodia have met, or that the BM is damaged or occluded by dead cells or debris. In all wound types and sizes, the lack of exposed BM causes lamellipodia to retract, which triggers contraction of the actomyosin cable (Figure 7A-4). Actomyosin contraction ensures close contact between the cells at the wound edges as the wound closes, and extrudes dead cells or cell debris from the gap (Figure 7A-5). This elegant, coordinated series of events ensures closure of the many kinds of wounds that could be created by injuries in nature.

The selection of healing mechanisms for wounds of different shapes and sizes, with/without occluding debris and with/without BM damage, can be represented as a decision tree (Figure 7B), with the availability of the BM as a critical node. While many mechanistic questions remain, this shows that, in Clytia, the complex cell behaviors and structures seen in different wounding scenarios can be understood as a predictable sequence of decisions and events that lead to optimal wound closure.

### Actomyosin contraction across multiple scales of wound healing

A key finding in this study is that the formation and contraction of an actomyosin structure is ubiquitous in all epithelial wound healing contexts in Clytia, although the mechanism for forming the structure differs between micro-wounds (actin rings) and small or large wounds (multicellular actin cables attached at AJs). The parallels between actin rings and multicellular actin cables in various types of wound healing have been recognized before. For example, other researchers have pointed out that single-cell wounds in the plasma membrane heal through contraction of an actin ring, and noted the similarity to actin purse-strings (Clark *et al.,* 2009; Sonnemann and Bement, 2011). (Note that the single-cell wounds referred to in these studies are holes in the cell surface, in contrast to micro-wounds described here in Clytia that pass through all cell membranes and create a hole that exposes the BM underlying the epithelial monolayer). Abreu-Blanco and colleagues (Abreu-Blanco, Watts, *et al.,* 2012) further noted the similarity of multicellular actin cables that form at the edge of large wounds and the “purse strings” that surround small circular wounds. Here, we extend these ideas to encompass wounds that pass all the way through a single cell as well as small and large intercellular gaps, showing that actomyosin contraction is involved in healing all these wound types. Our observations further suggest that actomyosin contraction is critical for stretching cells over regions of BM damage, where lamellipodia cannot persist, and for extruding debris that might otherwise prevent healing.

### Lamellipodia extension across multiple scales of wound healing

Lamellipodia formation is also ubiquitous in epithelial wound healing in Clytia. The triggers for lamellipodia formation in response to wounding have been intensively studied in a variety of systems. Loss of cell tension, loss of cell junctions, or the lack of constraint provided by a wound have all been suggested (Enyedi and Niethammer, 2015). Our study of micro-wounds within a single cell suggests that the dissolution of cell:cell junctions is not necessary to trigger membrane protrusions in Clytia, and instead supports the idea that the presence of an exposed BM is necessary and sufficient for stable lamellipodia formation. It is interesting that lamellipodia originating from the margins of a wound within a single cell fuse rather than forming cell:cell junctions, suggesting that the proteins involved in junction formation may not be targeted to these internal membrane projections.

In many systems, wounds have been reported to heal solely with actin cables. In contrast, in Clytia, lamellipodia are seen in all wound types unless the BM is damaged or occluded. The wounding protocols used in other studies may damage the BM, preventing stable lamellipodia formation and leading to the erroneous conclusion that lamellipodia are not normally part of wound healing in that system. Laser ablation seems particularly likely to create BM damage, and we have observed that wounds produced with lasers do not form lamellipodia in Clytia (data not shown). In addition, BMs may be aberrant or completely absent in epithelial monolayers in culture.

### Co-occurrence and cooperation of actomyosin cables and lamellipodia in wound healing—the critical role of the basement membrane

Since the discovery of the purse string (Martin and Lewis, 1992), various factors have been proposed to determine why the lamellipodia or purse string mechanism dominates in a particular wound, including the origin of the tissue and the geometry of the wound (Ravasio *et al.,* 2015; Begnaud *et al.,* 2016). Many studies have also described the cooperation between lamellipodia and purse strings in wound healing in epithelial cells (Klarlund, 2012; Abreu-Blanco, Verboon, *et al.,* 2012; Bement *et al.,* 1993; Wood *et al.,* 2002; Danjo and Gipson, 1998; Rothenberg and Fernandez-Gonzalez, 2019; Vedula *et al.,* 2014; summarized in Begnaud *et al.,* (2016)). However, the choice and temporal deployment pattern of these mechanisms across different scales and contexts is poorly understood, as is the interaction of the structures with each other. In few cases hascollective cell migration, seen in large wounds, been integrated into these models. Still more rare is the consideration of micro-wounds that form holes through a single cell.

Interpretation of our data unravels some of these mysteries in Clytia. First, our work shows that the formation and persistence of laemllipodia and actomyosin cables are independent in wounds at all spatial scales, as each exists and functions when the other is inhibited by drugs. Second, our imaging clearly shows the co-existence of lamellipodia and actin cables, not only at the level of the wounded tissue but also at the level of the individual cell. Similarly, in *ex vivo* corneas from a mouse, where Danjo and Gipson, 1998 removed the epithelial cells carefully, leaving the BM intact, lamellipodia and a multicellular actin cable were observed simultaneously at the wound edge, closely resembling our observations in Clytia Ravisio *et al.,* (2015) described the presence of both lamellipodia and purse strings surrounding an *in vitro* wound, and Rajakyla *et al*. (2020) studied vertebrate epithelial cells reaching confluency in tissue culture and reported the fusion of transverse arcs from the lamellipodia to form cables extending from lateral AJs while lamellipodia persisted. Third, our findings demonstrate that the actomyosin cable can assemble and persist in the absence of active contraction, thereby highlighting the regulation of cable contraction as a critical and distinct component of wound healing mechanisms. Finally, our work shows that there is a sequential nature to the deployment of the two major closure mechanisms in Clytia: lamellipodia extension is followed by actomyosin contraction. This likely allows the lamellipodia to explore and locate cellular partners across a wound gap, and then signal by retraction that the gap is either closed or occluded, thus triggering actomyosin contraction. The BM hence plays an under-appreciated role in orchestrating these events. When exposed, lamellipodia are stable and extend, but when the BM is unavailable, lamellipodia retract, and actomyosin contraction initiates. The existence of stable lamellipodia that are unable to extend and an exposed BM, uniquely seen in large wounds, induces collective cell migration. The specific mechanisms that link BM availability, lamellipodia extension/retraction, and actin cable activity will be exciting to explore.

### Signaling to induce collective cell migration

Triggers for collective cell migration have been extensively studied without a clear consensus. It has been shown that, even in coherent epithelial sheets, both marginal and submarginal cells participate in movement towards the wound gap (Poujade *et al.,* 2007; Wu and Wong, 2025; Farooqui and Fenteany, 2005; Fenteany *et al.,* 2000). An early signaling event is a wave of calcium that moves from the wound margin to the submarginal cells within minutes of wound formation (Enyedi and Niethammer, 2015; Niethammer, 2016; Di Virgilio *et al.,* 2020), while waves of myosin activation and other signaling pathways have also been reported (Razzell *et al.,* 2014; Matsubayashi *et al.,* 2011). Here, we demonstrate a wave of actin relocalization and membrane protrusive activity. Our experiments suggest that this wave is a response to cell stretching or movement, with rows of cells more proximal to the wounds activating the cells behind them. It is unclear whether the protrusions are the result of gaps created between cells by the pulling action or are more complex responses to other signaling waves.

We previously demonstrated that extracellular ATP (eATP) enhances communication from marginal to submarginal cells over short distances in Clytia (Lee, O'Malley-Krohn, *et al.,* 2023) and promotes wound closure. However, our experiments with CellMask do not support the idea that eATP released from damaged cells is the primary long-distance signal in submarginal cell activation. Nevertheless, it cannot be ruled out that different factors are released during wounding than in response to CellMask toxicity.

### Relevance across the tree of life

It is important to ask whether the decision tree that governs the deployment of different mechanisms in wound healing in Clytia is relevant to other animals, including more complex organisms, especially since the strategy of sequential lamellipodia formation and actomyosin contraction orchestrated by the availability of the BM has not been reported in other models. The reports of co-existing lamellipodia and actin cables in vertebrate tissues (Danjo and Gipson, 1998; Rajakylä *et al.,* 2020) and the well-documented participation of both lamellipodia and purse strings in wound healing in vertebrate epithelial cells (Klarlund, 2012; Abreu-Blanco, Verboon, *et al.,* 2012; Begnaud *et al.,* 2016; Bement *et al.,* 1993; Wood *et al.,* 2002; Danjo and Gipson, 1998; Vedula *et al.,* 2014) indicate that evolutionarily distant organisms face the same regulatory challenges in selecting between lamellipodial and purse string mechanisms of wound closure. However, many examples implicate very different decision nodes in other systems. For instance, wounds in mouse early embryos heal through purse string closure but at stage E15 transition to healing with lamellipodia (Takaya *et al.,* 2022), suggesting a changing developmental context. In coelomycetes of sea urchin, micro-wounds that go all the way through the lamellipodia and seal, similar to the micro-wounds we describe in Clytia, heal without forming an actomyosin cable (Henson *et al.,* 2002), perhaps providing an example of a tissue-specific wound-healing strategy. It is also reasonable to speculate that more complex regulatory mechanisms for sensing and responding to different types and sizes of wounds may have evolved over time. Despite these caveats, the Clytia model presented here demonstrates that we can predict how cellular behaviors are orchestrated in many wound healing contexts in one organism. This should lead to testable hypotheses in vertebrate and other systems, which in turn may reveal unsuspected conserved strategies and regulatory steps in optimizing epithelial wound healing.

## METHODS

### Animal husbandry

Clytia colonies are maintained as previously described (Kamran *et al.,* 2017; Lee, O'Malley-Krohn, *et al.,* 2023). Around 3–4-week-old males (Z-23) and females (Z-30) were used for all analyses. No differences were seen between healing in males and females.

### Wounding

Medusa were placed bell side up in glass depression slides. Wounds of various sizes were made as described in the text. For wounds made with CellMask plasma membrane stain (Fisher Scientific, Catalogue# C10046), medusa were incubated with 0.5X CellMask stain (1:2000 dilution in filter-sterile artificial sea water (FSW) for 15 min. After rinsing with FSW, one medusa was transferred to a depression slide, covered with a coverslip, and exposed to green light for 2 min under a 20x objective lens. After the light exposure, the animal was transferred to a cell strainer filled with FSW in a 35 × 10 mm plastic dish and, at the indicated time, fixed, permeabilized, and stained with phalloidin (see Phalloidin Staining).

### Imaging and image analysis

DIC imaging was done on a Leica DM microscope. Images of healing wounds were captured at 5 s intervals for micro-wounds and 11 s intervals for small wounds using ZEN software. CZI images were corrected for brightness/contrast using FIJI ImageJ software (Schindelin *et al.,* 2012) and saved as single images (TIFF) or time-lapse video files (AVI) as described (Lee *et al.,* 2023). For time-lapse videos, images were registered using the FIJI plugin Registration > Linear stack alignment with SIFT alignment with default settings. Fluorescent images of actin in large wounds were also collected on the Leica DM microscope. Images of phalloidin-stained tissues were obtained using a Zeiss LSM 800 confocal scanning microscope.

For quantifying actin relocalization in marginal and submarginal cells in large wounds, images were first corrected in FIJI using process>subtract background (rolling ball, 50 pixels). For each processed image, three rectangular regions perpendicular to the wound edge were randomly selected, and the number of marginal and submarginal cell rows with actin accumulated at the edge was counted using the multipoint tool of Fiji ImageJ. The average of the three cell row counts was calculated for each image.

### Phalloidin staining

For phalloidin staining of actin, 3–5 medusa were wounded and placed in a 35 × 10 mm plastic dish containing a cell strainer filled with sterile FSW. At the appropriate time point post-wounding, medusa were transferred in the strainer to a fresh dish with fixation solution (7.42 ml FSW, 2.5 ml 16% methanol-free PFA, and 81 ul 25% methanol-free Glutaraldehyde). Tissues were fixed for 5 min, rinsed in FSW, and permeabilized for 3 min in 0.1% Triton X-100 in FSW. For tissues co-stained with Cytoliner (Biotium, Catalogue#:30133-T), tissues were then transferred to 2 ml FSW with 20 ul Cytoliner buffer, and 1 ul Cytoliner stain for 10 min and rinsed before proceeding with phalloidin staining. Animals were transferred to a 1:5000 FSW dilution of Phalloidin-iFlour 488 Conjugate (Cayman Chemical, Catalogue#:20549) for 1 h, then rinsed and imaged.

### Pharmacology

Live animals were treated with inhibitors by incubating for 20 min in 5 ml of the inhibitor solution in small petri dishes. Latrunculin A (Cayman Chemical Catalogue#:10010630): 5 ul of solution supplied in ethanol was dried and resuspended in 5 ul of DMSO to create a stock. Latrunculin A high concentration: stock was diluted 1:10 in DMSO and then 1:1000 in FSW (final concentration 23.72nM). Latrunculin A low concentration: stock was diluted 1:16 in DMSO and then 1:1000 dilution in FSW (final concentration 14.23nM). Cytochalasin B (Cayman Chemical, Catalogue#:11328): a 1mM stock was prepared in DMSO and diluted 1:1000 in FSW (final concentration 1 µM). (-)-Blebbistatin (Cayman Chemical, Catalogue#: 13013-1): a 6.8mM stock was prepared in DMSO and diluted 1:1000 in FSW (final concentration 6.8µM). Appropriate dilutions of DMSO were used to treat control animals.

## Supporting information





Supporting Video 1 Movie S1 Closure of microwounds between and within epithelial cells. Note lamellipdia expension in both wound types. Little debris is apparent in wound gaps. Images were captured every 5 seconds. Frame rate 7fps.

Supporting Video 2 Movie S2 Closure of microwounds with debris in intracellular wounds. Note small lamellipodia extension followed by contraction to extrude debris. Images were captured every 5 seconds. Frame rate 7fps.

Supporting Video 3 Movie S3 Higher magnification of microwound healing shows fusion of intracellular lamellapodia and extrusion of debris. Images were captured every 5 seconds. Frame rate 7fps.

Supporting Video 4 Movie S4 Extrusion of debris from an intracellular wound. Images were captured every 5 seconds. Frame rate 7fps.

Supporting Video 5 Movie S5 Lack of lamellipodia, actin ring contraction, or healing of microwounds in animals treated with high concentration Latrunculin A. Images were captured every 5 seconds. Frame rate 7fps.

Supporting Video 6 Movie S6 Healing of microwounds by lamellipodia without actin ring contraction after Blebbistatin treatment. Images were captured every 5 seconds. Frame rate 7fps.

Supporting Video 7 Movie S7 Healing of microwounds by actin ring contraction without lamellipodia after Cytochalasin B treatment. Images were captured every 5 seconds. Frame rate 7fps.

Supporting Video 8 Movie S8 Healing of microwounds by actin ring contraction without lamellipodia after low concnetration Latrunculin A treatment. Images were captured every 5 seconds. Frame rate 7fps.

Supporting Video 9 Movie S9 Closure of a small intercellular wound showing lamellipodia extension followed by actomyosin cable contractions. Images were captured every 11 seconds. Frame rate 7fps.

Supporting Video 10 Movie S10 Lack of closure of small intercellular wounds after low concentration Latrunculin A treatment. Lamellipodia formation is inhibited. Some actomyosin contraction is seen at the perimeter but fails to close wound. Images were captured every 11 seconds. Frame rate 7fps.

Supporting Video 11 Movie S11 Lack of closure of small intercellular wounds after low concentration Cytochalasin B treatment. Lamellipodia formation is inhibited. Some actomyosin contraction is seen at the perimeter but fails to close wounds. Images were captured every 11 seconds. Frame rate 7fps.

Supporting Video 12 Movie S12 An actomyosin “purse string” contraction closes a wound where the basement membrane is damaged. The edge of the basement membrane can be seen at the lower right hand side of the wound. Images were captured every 11 seconds. Frame rate 7fps.

Supporting Video 13 Movie S13 An actomyosin “purse string” contraction extrudes a dead cell. Images were captured every 11 seconds. Frame rate 7fps.

Supporting Video 14 Movie S14 Collective cell migration in the process of closing a large wound. Images were captured every 11 seconds. Frame rate 7fps.

Supporting Video 15 Movie S15 Low magnification shows healing of a large wound. Collective cell migration allows marginal cell lamellipodia to meet across the wound gap, followed by an actomyosin cable contraction. Images were captured every 11 seconds.

## Abbreviations used:

AJs: adherens junctions
BM: basement membrane
Clytia: Clytia hemispaerica
LatA: latrunculinA.

## References

[B1] Abreu-Blanco MT, Verboon JM, Liu R, Watts JJ, Parkhurst SM (2012). *Drosophila* embryos close epithelial wounds using a combination of cellular protrusions and an actomyosin purse string. J Cell Sci 125, 5984–5997. 10.1242/jcs.109066. 23038780 PMC3585516

[B2] Abreu-Blanco MT, Watts JJ, Verboon JM, Parkhurst SM (2012). Cytoskeleton responses in wound repair. Cell Mol Life Sci 69, 2469–2483. 10.1007/s00018-012-0928-2. 22349211 PMC3388155

[B3] Anon E, Serra-Picamal X, Hersen P, Gauthier NC, Sheetz MP, Trepat X, Ladoux B (2012). Cell crawling mediates collective cell migration to close undamaged epithelial gaps. Proc Natl Acad Sci USA 109, 10891–10896. 10.1073/pnas.1117814109. 22711834 PMC3390890

[B4] Begnaud S, Chen T, Delacour D, Mège Ré-M, Ladoux Bît (2016). Mechanics of epithelial tissues during gap closure. Curr Opin Cell Biol 42, 52–62. 10.1016/j.ceb.2016.04.006. 27131272 PMC5428743

[B5] Bement WM, Forscher P, Mooseker MS (1993). A novel cytoskeletal structure involved in purse string wound closure and cell polarity maintenance. J Cell Biol 121, 565–78. 10.1083/jcb.121.3.565. 8486737 PMC2119560

[B6] Bornes L, Windoffer R, Leube RE, Morgner J, van Rheenen J (2021). Scratch-induced partial skin wounds re-epithelialize by sheets of independently migrating keratinocytes. Life Sci Alliance 4, e202000765. 10.26508/lsa.202000765. 33257474 PMC7723264

[B7] Casares L, Vincent R, Zalvidea D, *et al.* (2015). Hydraulic fracture during epithelial stretching. Nat Mater 14, 343–51. 10.1038/nmat4206. 25664452 PMC4374166

[B8] Clark AG, Miller AL, Vaughan E, Yu H-YE, Penkert R, Bement WM (2009). Integration of single and multicellular wound responses. Curr Biol 19, 1389–95. 10.1016/j.cub.2009.06.044. 19631537 PMC3561667

[B9] Gordon SR (2022). Dancing to a somewhat different rhythm: Cell migration along the natural basement membrane. Biocell 46, 2059–63. 10.32604/biocell.2022.019873.

[B10] Danjo Y, Gipson IK (1998). Actin ‘purse string’ filaments are anchored by E-cadherin-mediated adherens junctions at the leading edge of the epithelial wound, providing coordinated cell movement. J Cell Sci 111, 3323–32. 10.1242/jcs.111.22.3323. 9788874

[B11] Di Virgilio F, Sarti AC, Coutinho-Silva R (2020). Purinergic signaling, DAMPs, inflammation. Am J Physiol Cell Physiol 318, C832–35. 10.1152/ajpcell.00053.2020. 32159362

[B12] Enyedi Bá, Niethammer P (2015). Mechanisms of epithelial wound detection. Trends Cell Biol 25, 398–407. 10.1016/j.tcb.2015.02.007. 25813429 PMC4475481

[B13] Farooqui R, Fenteany G (2005). Multiple rows of cells behind an epithelial wound edge extend cryptic lamellipodia to collectively drive cell-sheet movement. J Cell Sci 118, 51–63. 10.1242/jcs.01577. 15585576

[B14] Fenteany G, Janmey PA, Stossel TP (2000). Signaling pathways and cell mechanics involved in wound closure by epithelial cell sheets. Curr Biol 10, 831–38. 10.1016/S0960-9822(00)00579-0. 10899000

[B15] Fernandez-Gonzalez R, Zallen JA (2013). Wounded cells drive rapid epidermal repair in the early *Drosophila* embryo. Mol Biol Cell 24, 3227–37. 10.1091/mbc.e13-05-0228. 23985320 PMC3806660

[B16] Friedl P, Wolf K, Zegers MM (2014). Rho-directed forces in collective migration. Nat Cell Biology 16, 208–10. 10.1038/ncb2923. 24576897

[B17] Haensel D, Dai X (2018). Epithelial-to-mesenchymal transition in cutaneous wound healing: Where we are and where we are heading. Developmental Dynamics : An Official Publication of the American Association of Anatomists 247, 473–80. 10.1002/dvdy.24561. 28795450 PMC5809211

[B18] Henson JH, Nazarian R, Schulberg KL, *et al.* (2002). Wound closure in the lamellipodia of single cells: Mediation by actin polymerization in the absence of an actomyosin purse string. Mol Biol Cell 13, 1001–14. 10.1091/mbc.01-04-0167. 11907278 PMC99615

[B19] Ivanov AI, Lechuga S, Marino-Melendez A, Naydenov NG (2022). Unique and redundant functions of cytoplasmic actins and nonmuscle Myosin II isoforms at epithelial junctions. Ann NY Acad Sci 1515, 61–74. 10.1111/nyas.14808. 35673768 PMC9489603

[B20] Jain P, Bernhard Rauer S, Möller M, Singh S (2022). Mimicking the natural basement membrane for advanced tissue engineering. Biomacromolecules 23, 3081–103. 10.1021/acs.biomac.2c00402. 35839343 PMC9364315

[B21] Kamran Z, Zellner K, Kyriazes H, Kraus CM, Reynier J-B, Malamy JE (2017). In vivo imaging of epithelial wound healing in the cnidarian Clytia hemisphaerica demonstrates early evolution of purse string and cell crawling closure mechanisms. BMC Dev Biol 17, 17. 10.1186/s12861-017-0160-2. 29258421 PMC5735930

[B22] Klarlund JK (2012). Dual modes of motility at the leading edge of migrating epithelial cell sheets. Proc Nat Acad Sci 109, 15799–804. 10.1073/pnas.1210992109. 23019364 PMC3465438

[B23] Lee EEL, O'Malley-Krohn I, Edsinger E, Wu S, Malamy J (2023). Epithelial wound healing in Clytia hemisphaerica provides insights into extracellular ATP signaling mechanisms and P2XR evolution. Sci Rep 13, 18819. 10.1038/s41598-023-45424-5. 37914720 PMC10620158

[B24] Lee EEL, Watto E, Malamy J (2023). Characterizing epithelial wound healing in vivo using the cnidarian model organism *Clytia hemisphaerica*. J Vis Exp (JoVE) 192, e65081. 10.3791/65081. 36847403

[B25] Li Li, He Y, Zhao M, Jiang J (2015). Collective cell migration: implications for wound healing and cancer invasion. Burns & Trauma 1, 21–26. 10.4103/2321-3868.113331. PMC4994501 27574618

[B26] Liang C-C, Park AY, Guan J-L (2007). In vitro scratch assay: A convenient and inexpensive method for analysis of cell migration in vitro. Nat Protoc 2, 329–33. 10.1038/nprot.2007.30. 17406593

[B27] Lu P, Lu Y (2021). Born to run? Diverse modes of epithelial migration. Front Cell Dev Biol 9, 704939. 10.3389/fcell.2021.704939. 34540829 PMC8448196

[B28] Martin P, Lewis J (1992). Actin cables and epidermal movement in embryonic wound healing. Nature 360, 179–83. 10.1038/360179a0. 1436096

[B29] Matsubayashi Y, Ebisuya M, Honjoh S, Nishida E (2004). ERK Activation Propagates in Epithelial Cell Sheets and Regulates Their Migration during Wound Healing. Curr Biol 14, 731–35. 10.1016/j.cub.2004.03.060. 15084290

[B30] Matsubayashi Y, Razzell W, Martin P (2011). ‘White wave’ analysis of epithelial scratch wound healing reveals how cells mobilise back from the leading edge in a Myosin-II-dependent fashion. J Cell Sci 124, 1017–21. 10.1242/jcs.080853. 21402875 PMC3056603

[B31] Niethammer P (2016). The early wound signals. Curr Opin Genet Dev 40, 17–22. 10.1016/j.gde.2016.05.001. 27266971 PMC5278878

[B32] Ozawa M, Hiver S, Yamamoto T, *et al.* (2020). Adherens junction regulates cryptic lamellipodia formation for epithelial cell migration. J Cell Biol 219, e202006196. 10.1083/jcb.202006196. 32886101 PMC7659716

[B33] Park S, Gonzalez DG, Guirao B, *et al.* (2017). Tissue-scale coordination of cellular behaviour promotes epidermal wound repair in live mice. Nat Cell Biology 19, 155–63. 10.1038/ncb3472. 28248302 PMC5581297

[B34] Pastar I, Stojadinovic O, Yin NC, *et al.* (2014). Epithelialization in wound healing: A comprehensive review. Adv Wound Care 3, 445–64. 10.1089/wound.2013.0473. PMC4086220 25032064

[B35] Poujade M, Grasland-Mongrain E, Hertzog A, *et al.* (2007). Collective migration of an epithelial monolayer in response to a model wound. Physical Sciences. Proceedings of the National Academy of Sciences 104, 15988–93. 10.1073/pnas.0705062104. PMC2042149 17905871

[B36] Rajakylä EK, Lehtimäki JI, Acheva A, *et al.* (2020). Assembly of peripheral actomyosin bundles in epithelial cells is dependent on the CaMKK2/AMPK pathway. Cell Rep 30, 4266–4280.e4. 10.1016/j.celrep.2020.02.096. 32209483

[B37] Ravasio A, Cheddadi I, Chen T, *et al.* (2015). Gap geometry dictates epithelial closure efficiency. Nat Commun 6, 7683. 10.1038/ncomms8683. 26158873 PMC4510701

[B38] Razzell W, Wood W, Martin P (2014). Recapitulation of morphogenetic cell shape changes enables wound re-epithelialisation. Development 141, 1814–20. 10.1242/dev.107045. 24718989 PMC3994776

[B39] Richardson R, Metzger M, Knyphausen P, *et al.* (2016). Re-epithelialization of cutaneous wounds in adult zebrafish combines mechanisms of wound closure in embryonic and adult mammals. J Cell Sci 129, e1.1–e1.1. 10.1242/jcs.193763. PMC4920168 27122176

[B40] Rothenberg KE, Fernandez-Gonzalez R (2019). Forceful closure: Cytoskeletal networks in embryonic wound repair. Mol Biol Cell 30, 1353–58. 10.1091/mbc.E18-04-0248. 31145669 PMC6724689

[B41] Schindelin J, Arganda-Carreras I, Frise E, *et al.* (2012). Fiji: An open-source platform for biological-image analysis. Nat Methods 9, 676–82. 10.1038/nmeth.2019. 22743772 PMC3855844

[B42] Sonnemann KJ, Bement WM (2011). Wound repair: toward understanding and integration of single-cell and multicellular wound responses. Annu Rev Cell Dev Biol 27, 237–63. 10.1146/annurev-cellbio-092910-154251. 21721944 PMC4878020

[B43] Takaya K, Okabe K, Ishigami A, *et al.* (2022). Actin cable formation and epidermis–dermis positional relationship during complete skin regeneration. Sci Rep 12, 15913. 10.1038/s41598-022-18175-y. 36151111 PMC9508246

[B44] Tanner K, Ferris DR, Lanzano L, *et al.* (2009). Coherent movement of cell layers during wound healing by image correlation spectroscopy. Biophys J 97, 2098–106. 10.1016/j.bpj.2009.06.052. 19804742 PMC2756390

[B45] Theveneau E, Mayor R (2013). Collective cell migration of epithelial and mesenchymal cells. Cell Mol Life Sci CMLS 70, 3481–92. 10.1007/s00018-012-1251-7. 23314710 PMC11113167

[B46] Tsai C-R, Wang Y, Galko MJ (2018). Crawling wounded: molecular genetic insights into wound healing from drosophila larvae. Int J Dev Biol 62, 479–89. 10.1387/ijdb.180085mg. 29938760 PMC6352908

[B47] Tyler S (2003). Epithelium—The primary building block for metazoan complexity1. Integr Comp Biol 43, 55–63. 10.1093/icb/43.1.55. 21680409

[B48] Vedula SRK, Hirata H, Nai MH, *et al.* (2014). Epithelial bridges maintain tissue integrity during collective cell migration. Nat Mat 13, 87–96. 10.1038/nmat3814. 24292420

[B49] Vedula SRK, Ravasio A, Lim CT, Ladoux B (2013). Collective cell migration: A mechanistic perspective. Physiology 28, 370–79. 10.1152/physiol.00033.2013. 24186932

[B50] Vicente-Manzanares M, Ma X, Adelstein RS, Horwitz AR (2009). Non-muscle myosin II takes centre stage in cell adhesion and migration. Nat Rev Mol Cell Biol 10, 778–90. 10.1038/nrm2786. 19851336 PMC2834236

[B51] Wood W, Jacinto A, Grose R, *et al.* (2002). Wound healing recapitulates morphogenesis in *Drosophila* embryos. Nat Cell Biology 4, 907–12. 10.1038/ncb875. 12402048

[B52] Wu Z, Wong M (2025). Collective cell migration across scales: A systems perspective. Semin Cell Dev Biol 173, 103628. 10.1016/j.semcdb.2025.103628. 40633502

[B53] Xu S, Chisholm AD (2011). A Gαq - Ca2+ signaling pathway promotes actin-mediated epidermal wound closure in *C. Elegans*. Curr Biol : CB 21, 1960–67. 10.1016/j.cub.2011.10.050. 22100061 PMC3237753

[B54] Zhao M, Song B, Pu J, Forrester JV, McCaig CD (2003). Direct visualization of a stratified epithelium reveals that wounds heal by unified sliding of cell sheets. FASEB J 17, 397–406. 10.1096/fj.02-0610com. 12631579 PMC1459285

