# Supplementary material for: The basement membrane determines the choice of wound healing mechanism across wound scales in the basal eukaryote Clytia hemisphaerica
Source: Mol Biol Cell. 2026 May 13;37(7):ar60. doi: 10.1091/mbc.E26-02-0094 (PMC13329811; doi:10.1091/mbc.E26-02-0094)
Supplement: Supplementary file 1 [file mbc-37-ar60-s001.pdf]

Supplemental Materials

Molecular Biology of the Cell

Malamy *et al*.

Figure S1

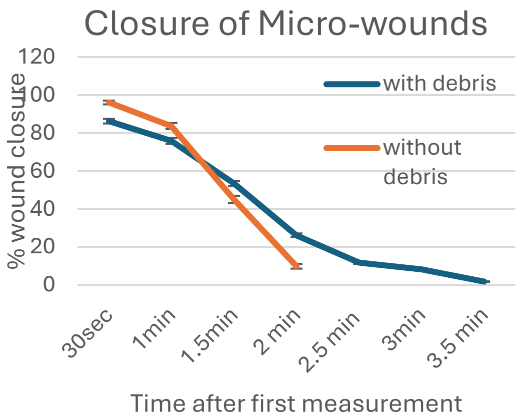

Figure S2

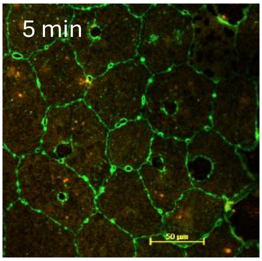

Figure S3

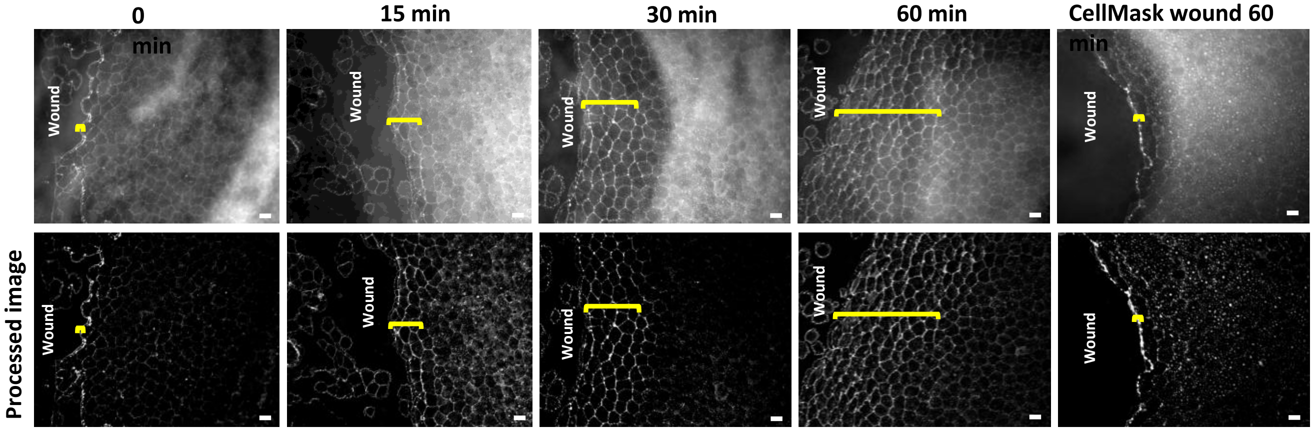

Figure S1 Closure of micro-wounds with debris is slower than in wounds without debris. Perimeters of 10 wounds were traced manually. The outermost perimeter of the wound was measured, which did not take into account the coverage of the gap by lamellipodia. Wounds with debris were all within cells, while those without debris were within or between cells. Points indicate average reduction in wound area compared to T0 (%, n=10). Error bars = standard error.

Figure S2 Cytochalasin B treatment does not inhibit actin ring formation. Actin rings are apparent 5 minutes after creating micro-wounds in Cytochalasin B treated tissues.

Figure S3 Image processing to better identify the boundary between submarginal cells with and without relocalized actin. Raw (top row) and processed (bottom row) images of phalloidin-stained actin in large wounds at various time points are shown.
