# Supplementary material for: The basement membrane determines the choice of wound healing mechanism across wound scales in the basal eukaryote Clytia hemisphaerica
Source: Mol Biol Cell. 2026 May 13;37(7):ar60. doi: 10.1091/mbc.E26-02-0094 (PMC13329811; doi:10.1091/mbc.E26-02-0094)
Supplement: Supplementary file 17 [file reviewer_reportE26-02-0094.pdf]

The basement membrane determines the choice of wound healing mechanism across wound scales in the basal eukaryote Clytia hemiphaerica

Jocelyn Malamy, Maxwell Sassaman, and Manjula Mony

Corresponding author(s): Jocelyn Malamy, University of Chicago

**Review Timeline:**	Submission Date:	2026-02-25
	Editorial Decision:	2026-04-05
	Revision Received:	2026-04-28
	Accepted:	2026-05-04

Editor-in-Chief: John Aitchison

Transaction Report:

(Note: With the exception of the correction of typographical or spelling errors that could be a source of ambiguity, letters and reports are not edited. The original formatting of letters and referee reports may not be reflected in this compilation.)

RE: Manuscript #E26-02-0094
TITLE: A single strategy describes healing of all epithelial wound types in the basal eukaryote Clytia hemisphaerica

Dear Dr. Malamy,

Your manuscript, "A single strategy describes healing of all epithelial wound types in the basal eukaryote Clytia hemisphaerica" has now been evaluated by two experts. As you will see, they differ in their opinion as to the suitability of this study for publication in MBoC, with reviewer 1 having only one minor concern and reviewer 2 having several major concerns. However, a close inspection of the comments of reviewer 2 as well as your manuscript leads me to believe that nearly all of reviewer 2's comments can be addressed by textual revisions. Accordingly, we would be willing to consider a revised manuscript that addresses each of the points below.

1. In reference to the various processes you have reported as being involved in wound repair, Reviewer 2 writes "It is hard to understand how these different mechanisms are providing a single set of rules or single strategy for wound repair or what that means in terms of the repair process".

The issue here seems to be one of wording. Presumably, you do not mean that there is no difference between multicellular purse strings, lamellipodia and collective cell migration and yet this is what is implied by your title and the language you use at several points in this manuscript. Moreover, you also point out that the healing response differs in the presence and absence of the basement membrane which surely implies that there is more than one "rule" or "strategy". We therefore ask that you rework the title and the relevant places in the text to avoid giving the impression that only a single mechanism, strategy or rule is in play. Possibilities include referring to lamellipodia and purse strings being used in a consistent fashion across different wound scales. You may also want to incorporate the basement membrane into the title as that will increase the interest in your study, but that is up to you.

2. Reviewer 2 points out that it is not clear how many experiments your findings are based on and that there is insufficient quantification. This concern can be addressed by including quantifications as well as statistical analyses for any quantitative claims. In some cases where claims are qualitative or difficult to quantify, it may be sufficient to state that results shown are representative of X number of experiments; failing this, such claims should be dropped.

3. Reviewer 2 raises the question of the potential impact of wound size and shape on the interpretation of the results. This concern can be addressed by acknowledging that the wounds vary in terms of size and shape and by discussing whether these differences have any apparent impact on the results.

4. Similar to point 3, Reviewer 2 notes that there are varying numbers of wounds produced in the experiments used to generate "microwounds" (ie full-thickness wounds of individual cells) and wants to know if the healing process differs as a function of nearness to other wounds. This concern can also be addressed by acknowledging that the number of wounds varies and by discussing whether this variation impacts the reported results.

5. Reviewer 2 points out that there is good evidence that there is fundamental variation in how healing occurs in different model systems. For that reason, Reviewer 2 feels that claims of universality based on the findings presented here are an overstatement. This concern can be addressed by toning down or removing claims of universality (see also below).

6. My impression after reading your study is that you could strengthen its impact with revisions that increase clarity and reduce the potential for misunderstandings about previous work. Some of this will be accomplished by addressing point 1 above. For example, it took several readings and repeated viewing of Figure 7 before your main point sank in. Additionally, it would be to your advantage to focus on what is truly new here. For example, the statement "The co-existence of lamellipodia and actomyosin structures surrounding wounds is counter to the general assumption that cells will have one or the other of these actin-based structures driving wound healing" might lead readers to believe that you consider this one of the novel findings of your study. However, the dynamic coexistence of purse strings and lamellipodia in the same wound was reported for healing of cultured human cell monolayers more than 30 years ago (pmid 8486737) and for Drosophila embryos more than 20 years ago (pmid 12402048). Thus, setting up lamellipodia and purse strings as "either/or" seem like a strawman to those familiar with the wound healing literature. Moreover, a little later in the Discussion, it is made clear that this really is a strawman when the reader is told " â€¦ the implication of cooperation between lamellipodia and purse strings in wound healing in vertebrate epithelia cells [3], [4], [5], [6], [7], [11], [50] suggests that the universal strategy for epithelial wound healing uncovered in Clytia may be widely conserved". Fortunately, this problem could be easily fixed by replacing the "either or" construct with a simple statement to the effect that while wounds have long been known to use purse strings and lamellipodia simultaneously to drive healing, the temporal deployment pattern of these mechanisms across different scales and conditions is poorly understood.

Another area of the text that seems likely to be misunderstood is the difference between the microwounds in this study and single cell wounds in most other studies. The microwounds in this study are apparently not holes in the cell surface on one side of the cell, but full thickness wounds such that the hole is surrounded on all sides by intact plasma membranes. Because of the

growing popularity of single cell repair as an object of study, this point should probably be emphasized in the discussion when microwounds are first discussed. Additionally, a key report in which similar full thickness wounds were studied is missing from the Discussion. That is, Henson et al. (2002; pmid 11907278) studied the healing of full thickness wounds in sea urchin coelomocytes. In that study the authors reported that such holes heal solely by lamellipodial extension with no evidence for purse strings. Why the results of that study differ from those of the current study is not clear, but this point needs to be addressed. Moreover, the results of that study (taken together with the comments of Reviewer 2) underscore the potential danger of overstating the generality of the findings of this study.

Finally, many thanks for submitting this fascinating study to MBoC. I look forward to seeing a revised manuscript.

Sincerely,

Bill Bement
Monitoring Editor
Molecular Biology of the Cell

Dear Dr. Malamy,

The review of your manuscript, referenced above, is now complete. The Monitoring Editor has decided that your manuscript is not acceptable for publication at this time, but may be deemed acceptable after specific revisions are made, as described in the Monitoring Editor's decision letter above and the reviewer comments below.

A reminder: Please do not contact the Monitoring Editor directly regarding your manuscript. If you have any questions regarding the review process or the decision, please contact the MBoC Editorial Office (mboc@ascb.org).

When submitting your revision include a rebuttal letter that details, point-by-point, how the Monitoring Editor's and reviewers' comments have been addressed. (The file type for this letter must be "rebuttal letter"; do not include your response to the Monitoring Editor and reviewers in a "cover letter.") Please bear in mind that your rebuttal letter will be published with your paper if it is accepted, unless you have opted out of publishing the review history.

Authors are allowed 90 days to submit a revision. If this time period is inadequate, please contact us at mboc@ascb.org.

Revised manuscripts are assigned to the original Monitoring Editor whenever possible. However, special circumstances may preclude this. Also, revised manuscripts are often sent out for re-review, usually to the original reviewers when possible. The Monitoring Editor may solicit additional reviews if it is deemed necessary to render a completely informed decision.

In preparing your revised manuscript, please follow the instruction in the Information for Authors (www.molbiolcell.org/info-for-authors). In particular, to prepare for the possible acceptance of your revised manuscript, submit final, publication-quality figures with your revision as described.

To submit the rebuttal letter, revised manuscript, and figures, use this link: Link Not Available

MBoC offers the option to publish your work with immediate open access. Open access can increase the discoverability and usability of your research. If you would like to publish your paper with immediate open access but did not select this option during initial submission, please contact the editorial office at mbc@ascb.org.

Authors of Articles and Brief Communications are also encouraged to create a short video abstract to accompany their article when it is published. These video abstracts, known as Science Sketches, are up to 2 minutes long and will be published on YouTube and then embedded in the article abstract. Science Sketch Editors on the MBoC Editorial Board will guide you as you prepare your video. Information about how to prepare and submit a video abstract is available at www.molbiolcell.org/science-sketches. Please contact mboc@ascb.org if you are interested in creating a Science Sketch.

Sign up for MBoC's new content alerts here https://tinyurl.com/3bcsyk3e

Thank you for submitting your manuscript to MBoC. We look forward to receiving your revised paper.

Sincerely,

Eric Baker
Journal Production Manager
MBoC Editorial Office

mbc@ascb.org

Reviewer #1 (Remarks to the Author):

Different studies of epithelial wound healing have revealed a variety of apparent mechanisms for closing the wound ranging from cell migration to trans-cellular actin purse strings. This has left the wound healing field in a somewhat confusing state since it seems like different systems use pretty much entirely different mechanical strategies to close the wound. However, the authors of this paper make the point, which I find compelling, that the wide variety of model systems and types of wounds (size, shape, method of creating, etc.) have made it actually hard to know whether the different systems really do close wounds differently, or whether instead it is just that when wounds are created using different modalities, the response is dominated by one type of closure mechanism versus another. The key of this paper is using a single model system (Clytia medusa) to study wounds at three different spatial scales, and the remarkable overall result is that at all three scales, both lamellipodial extension and actin cable contraction seem to play roles. I feel that the results are extremely interesting and important, and well documented, such that this paper is suitable for publication in MBoC. I have a couple of clarification suggestions that I would suggest the reviewers consider, that I think could help make the work easier to understand by readers and thereby increase the potential impact.

The demonstration that when a hole is punched in a cell it can form lamellipodia to span the hole is really remarkable and novel. They further show evidence that these internal lamellipodia somehow avoid forming junctions. I think these are both extremely interesting findings in basic cell biology that has ramifications about self-organization of leading edge structures and the recognition mechanisms involved in forming cell-cell junctions.

Given how interesting and important the results with micro-wounds are, I feel that it is important to have a little more information about how they are formed in order to know how to interpret the results. Mechanical pressure is used, but exactly what is it doing? Is it that the pressure applied perpendicular to the plane of the sheet produces a stretching in the plane of the sheet, i.e. via a Poisson ratio? Or does the pressure push the cell membrane on the top and the bottom of the cell together such that the membranes fuse together and create a donut-shaped cell? These could be very different mechanisms in that one would create a membrane rupture while the other might not. If I understand correctly, the authors are asserting the former explanation, i.e. stretching caused by deformation of the mesoglea, but they use the term "rebounds" which I find confusing since that sounds like it is something that happens after the pressure is removed. I think at the very least it would be important to clarify which direction the forces are acting. The images show cell debris, or at any rate they show a bunch of small round objects in the hole, but whether those came from inside the cell versus were created by some kind of membrane fusion event is a little hard to tell. I wonder whether it might be feasible to add a membrane-impermeant dye and show that it is entering the cells when they are compressed to make a wound?

Besides the micro-wounds, the finding of sequential deployment of lamellipodial extension and actin purse string contraction is very striking. By itself, this result could just mean that there is a rigidly programmed sequence of events, but the further experiments showing that when lamellipodial crawling is blocked, actin contraction takes over, provides good evidence that both mechanisms are working towards the same "goal" of closing the wound such that one can take over when the other isn't working.

One small question I had about the actin contraction mechanism, is that in many of the iamges, it did not really seem like the multicellular actin cable was surrounding the wound, but was only on one side or the other. (as in Figure 4C). If actin is only on one side, is it really able to create a contractile force that would close the wound? It would seem like it might just pinch one side. I think the authors should comment a bit about this. Maybe this is something that is well known in the literature, if so, citations should be provided. Also, in the example image, it seemed like the actin cable was forming on the concave side of the wound but not the convex side. If this is a general correlation, I think the authors should comment on that since it might explain why the cable only forms in certain regions. Moreover, restriction of the contractile cable to portions of the wound margin that are concave might be a mechanism by which even partial cables could lead to overall reduction of wound margin.

Reviewer #2 (Remarks to the Author):

In this manuscript, the authors examined epithelial wound healing at different scales in Clytia. First, the authors show that wounds generated within cells and between cells by applying pressure with a cover glass lead to cells exhibiting lamellipodia-like actin-rich structures within the wound and then forms an actin ring at the wound edge. Both structures are not formed in the presence of F-actin inhibitors, whereas actin is still enriched at the wound edge, suggesting that actin polymerization is not essential for the recruitment of actin to the wound edge. While the wound still closes when the contraction of the actin ring is

inhibited by Myosin II inhibitor, the cell debris is not removed from the wound site. On the other hand, inhibiting lamellipodia formation does not prevent the formation of the actin ring, and the wound is completely healed. Hence, the actomyosin ring formation is independent of lamellipodia formation and required to remove the cell debris after wound closure, but is not necessary for wound closure. Next, the authors examined different sizes of wounds in epithelial cells. Upon 1-3 cell-width wounding, adjacent cells at the wound form lamellipodia and a multicellular actin cable. The multicellular actin cable is not formed in the presence of a Myosin II inhibitor, suggesting that adherens junctions are required for its formation. When the basement membrane is damaged or a cell is dead, actomyosin contraction occurs without the formation of lamellipodia to heal the wound or remove a dead cell. Thus, the authors speculate that the lack of lamellipodia formation is the trigger for actomyosin cable contraction. Next, the authors examined repair dynamics in larger wounds. As observed in small multicellular wounds, lamellipodia and multicellular actin cables form at the wound edge, while actin cable contraction was not seen until a large gap was filled with lamellipodia through collective cell migration. During collective cell migration, cells adjacent to the wound exhibit enriched actin on the side of the wound. This actin enrichment spreads to ~10 cells by 60 min after wounding. This actin enrichment in submarginal cells is not observed in massive dead cells. Thus, diffusion signals from dead or damaged cells do not likely transfer information to neighboring cells.
This manuscript provides a description of what happens at different scales of wound healing in Clytia - which has several interesting features. However, the presented data do not fully support the authors' statements, especially regarding the proposed universal mechanisms in epithelial wound repair.

Major comments:

(1) The authors describe different mechanisms that are required to close wounds in Clytia. Actomyosin ring contraction, not the lamellipodia extension, is required for micro wounds and small wounds with basement membrane damage. Both lamellipodia extension and actomyosin ring contraction are required for small wound closure without basement membrane damage. Collective cell migration is required for larger wound closure. These results suggest that multiple mechanisms are used to repair wounds of different sizes in Clytia. It is hard to understand how these different mechanisms are providing a single set of rules or single strategy for wound repair or what that means in terms of the repair process.

(2) The descriptions presented here for wound repair in Clytia do not reach the level of those already published for other wound repair models. Robust quantification is required to support the authors' statement. Many conclusions in this manuscript rely on n=1 images with assumptions and without robust quantification. The images provided are not compelling enough to support the authors' statement, especially regarding the fluorescent images.
For example, in Figure 3, the authors state that actin exhibits the persistence of punctate patterns around wound gaps with debris at 5min. However, although those patterns are observed at 2.5 minutes, they do not appear at 1.5 and 3.5 minutes. Additionally, debris inside the wound is visible at 3.5 and 5 minutes, but not at 30 seconds, 1.5 minutes, or 2.5 minutes. The wound size at 1.5 minutes is smaller than at 2.5 and 3.5 minutes. In Figure 1, the wound size at 1.5 minutes is also smaller than at 2.5 minutes. How often are wounds with debris observed in different conditions, including drug treatment, and at different time points? Why does debris appear at later time points in the presence of blebbistatin, either because the wounds cannot close or because another mechanism, such as a membrane patch model, is involved in the repair process?
In Figure 4, the authors state that actomyosin contraction cannot fully close small wounds, but can close very small wounds. However, it is difficult to assess whether actin cables normally form in the presence of drugs, but cannot contract, or whether changing cell volume simply causes wounds to contract or fill the space regardless of actomyosin cable formation.
In Figure 6, the authors use dead cells to address whether a damage-induced diffusible signal is involved in the flow of information from marginal to submarginal cells. However, there is no citation showing that dead cells release the same damage-induced diffusible molecules when organisms are damaged to trigger wound healing processes. In addition, no phalloidin staining is shown. Additional data, higher quality images, and robust quantification are needed to support the stated conclusions.

(3) It is not clear to what degree the effects of different sizes and shapes of wounds are taken into account. Previous studies showed that repair mechanisms differ between small and large wounds (PMIDs: 24482116 and 35736340). Wounding methods in this manuscript result in a wide range of sizes and shapes at different scales. In particular, the drugs used in this study affect the cytoskeleton, thereby altering cell stiffness. In Figure 2D, all cells look like they are dissociating from each other, and a cell with low LatA is almost split in half. However, DIC images in 2A and 2B do not look like that. In addition, the shape of the wound is a factor in determining whether lamellipodia crowding or contractile ring formation during epithelial gap repair (PMID: 26158873). Indeed, all wounds are longitudinal shapes in Figure 4, whereas the wound shape in Figure 5 is rounded.

(4) The wounds generated are highly variable in the number and types of wounds. Do wounds within a cell heal with the same dynamics when it is attached to its neighboring cells compared to cases where many of the cells have been ripped apart from each other?

(5) Diverse mechanisms proposed in different models need to be considered. Previous studies using different models and wounding methods have suggested that organisms have distinct mechanisms for responding to various sizes and types of wounds to heal efficiently. Even in similar contexts of wound healing, different mechanisms have been proposed previously. For example, while actomyosin ring translocation is highly conserved for cell wound closure, the mechanisms of translocation are not identical and include Myosin-dependent contraction in the Drosophila embryo model, actin treadmilling in the Xenopus oocyte model, and Arp2/3-dependent actin assembly in the C. elegans model. The author's proposal that Clytia serves as a universal

model for all wound repair is not supported by their data and is an overstatement.

We are grateful to both reviewers for their insightful comments, and especially to the handling editor for his very clear guidance. Below we will address the comments of each reviewer point by point, followed by our response to the editor. Specific description of changes or additions are underlined in our responses and altered text is highlighted in yellow.

RE: Manuscript #E26-02-0094
TITLE: A single strategy describes healing of all epithelial wound types in the basal eukaryote Clytia hemisphaerica

Reviewer #1 (Remarks to the Author):

Different studies of epithelial wound healing have revealed a variety of apparent mechanisms for closing the wound ranging from cell migration to trans-cellular actin purse strings. This has left the wound healing field in a somewhat confusing state since it seems like different systems use pretty much entirely different mechanical strategies to close the wound. However, the authors of this paper make the point, which I find compelling, that the wide variety of model systems and types of wounds (size, shape, method of creating, etc.) have made it actually hard to know whether the different systems really do close wounds differently, or whether instead it is just that when wounds are created using different modalities, the response is dominated by one type of closure mechanism versus another. The key of this paper is using a single model system (Clytia medusa) to study wounds at three different spatial scales, and the remarkable overall result is that at all three scales, both lamellipodial extension and actin cable contraction seem to play roles. I feel that the results are extremely interesting and important, and well documented, such that this paper is suitable for publication in MBoC. I have a couple of clarification suggestions that I would suggest the reviewers consider, that I think could help make the work easier to understand by readers and thereby increase the potential impact.

The demonstration that when a hole is punched in a cell it can form lamellipodia to span the hole is really remarkable and novel. They further show evidence that these internal lamellipodia somehow avoid forming junctions. I think these are both extremely interesting findings in basic cell biology that has ramifications about self-organization of leading edge structures and the recognition mechanisms involved in forming cell-cell junctions.

We thank the reviewer for appreciating the novelty of this result and emphasizing that it is a key takeaway. To reflect this comment we now state in the abstract: Furthermore, the exposure of the basement membrane acts as a central regulatory cue, orchestrating lamellipodia formation, actomyosin contraction and collective cell migration responses.

Remarkably, we discovered that these same mechanisms operate in healing micro-wounds internal to a single cell.

Given how interesting and important the results with micro-wounds are, I feel that it is important to have a little more information about how they are formed in order to know how to interpret the results. Mechanical pressure is used, but exactly what is it doing? Is it that the pressure applied perpendicular to the plane of the sheet produces a stretching in the plane of the sheet, i.e. via a Poisson ratio? Or does the pressure push the cell membrane on the top and the bottom of the cell together such that the membranes fuse together and create a donut-shaped cell? These could be very different mechanisms in that one would create a membrane rupture while the other might not. If I understand correctly, the authors are asserting the former explanation, i.e. stretching caused by deformation of the mesoglea, but they use the term "rebounds" which I find confusing since that sounds like it is something that happens after the pressure is removed. I think at the very least it would be important to clarify which direction the forces are acting. The images show cell debris, or at any rate they show a bunch of small round objects in the hole, but whether those came from inside the cell versus were created by some kind of membrane fusion event is a little hard to tell. I wonder whether it might be feasible to add a membrane-impermeant dye and show that it is entering the cells when they are compressed to make a wound?

In terms of the mechanism of wound formation after the coverslip is dropped onto the bell, we agree with the reviewer that the forces that create the micro-wounds are unclear. It is apparent when doing these experiments that the whole animal is slightly squashed by the procedure despite the use of a depression slide, as it spreads out flat and covers a greater area of the slide upon addition of the coverslip. In videos we can clearly see that the cells are stretched to larger areas immediately after cover slip addition; we also see that the stretched cells then return to smaller areas. This leads us to believe that the stretching of the sheet leads to the observed tears (directly or indirectly), that the sheet does not remain stretched, and that perhaps the pressure of the coverslip compresses the animal but is pushed slightly upward by the rebound of the compressed mesoglea. Interestingly, Casares et al. (2015) studied epithelial cells growing on hydrogels, which closely resembles our epithelial cells growing on the mesoglea, and found that the highest tension on the sheet occurred not during stretching but when the hydrogel "rebounds" from stretching.

In sum, we can not say with certaintly whether the stretching of the sheet by the coverslip or the pressure during wounding or from the re-expanding mesoglea is responsible for the microwounds. We have changed the wording in the first paragraph of the results section to more clearly indicate this uncertainty:

"While the forces responsible for these wounds were not investigated, they likely result from the stretching of the epithelial sheet, compression of the cells and/or rebounding of the decompressed mesoglea ("jelly") after the initial pressure [37]."

While this is not completely satisfying, we don't believe that it greatly effects the interpretation of our results.

Besides the micro-wounds, the finding of sequential deployment of lamellipodial extension and actin purse string contraction is very striking. By itself, this result could just mean that there is a rigidly programmed sequence of events, but the further experiments showing that when lamellipodial crawling is blocked, actin contraction takes over, provides good evidence that both mechanisms are working towards the same "goal" of closing the wound such that one can take over when the other isn't working.

We absolutely agree with this assessment, and hopefully have made the model even more clear with our edits throughout the text and our addition of a Figure 7B decision tree.

One small question I had about the actin contraction mechanism, is that in many of the iamges, it did not really seem like the multicellular actin cable was surrounding the wound, but was only on one side or the other. (as in Figure 4C). If actin is only on one side, is it really able to create a contractile force that would close the wound? It would seem like it might just pinch one side. I think the authors should comment a bit about this. Maybe this is something that is well known in the literature, if so, citations should be provided. Also, in the example image, it seemed like the actin cable was forming on the concave side of the wound but not the convex side. If this is a general correlation, I think the authors should comment on that since it might explain why the cable only forms in certain regions. Moreover, restriction of the contractile cable to portions of the wound margin that are concave might be a mechanism by which even partial cables could lead to overall reduction of wound margin.

We have not observed a consistent trend of cables forming on only one side of the wound, or preferentially on the concave side. The reviewer raises an interesting point about uneven contraction on subsections of a wound margin, which we do regularly see. However, note that the small wounds shown in Figure 4 will seal through extension and adherence of the lamellipdial sides and fronts, and therefore it is unlikely that inconsistencies in the cable would make the closure fail. Indeed, the "liquidity" of lamellipodia may ensure that all areas of uneven wound gaps are covered. As we say in the discussion:

" Finally, our work shows that there is a sequential nature to the deployment of the two major closure mechanisms: lamellipodia extension is followed by actomyosin contraction. This likely allows the lamellipodia to explore and locate cellular partners across a wound gap, and then signal by retraction that the gap is either closed or occluded, thus triggering actomyosin contraction.."

Reviewer #2 (Remarks to the Author):

In this manuscript, the authors examined epithelial wound healing at different scales in Clytia. First, the authors show that wounds generated within cells and between cells by applying pressure with a cover glass lead to cells exhibiting lamellipodia-like actin-rich structures within the wound and then forms an actin ring at the wound edge. Both structures are not formed in the presence of F-actin inhibitors, whereas actin is still enriched at the wound edge, suggesting that actin polymerization is not essential for the recruitment of actin to the wound edge. While the wound still closes when the contraction of the actin ring is inhibited by Myosin II inhibitor, the cell debris is not removed from the wound site. On the other hand, inhibiting lamellipodia formation does not prevent the formation of the actin ring, and the wound is completely healed. Hence, the actomyosin ring formation is independent of lamellipodia formation and required to remove the cell debris after wound closure, but is not necessary for wound closure. Next, the authors examined different sizes of wounds in epithelial cells. Upon 1-3 cell-width wounding, adjacent cells at the wound form lamellipodia and a multicellular actin cable. The multicellular actin cable is not formed in the presence of a Myosin II inhibitor, suggesting that adherens junctions are required for its formation. When the basement membrane is damaged or a cell is dead, actomyosin contraction occurs without the formation of lamellipodia to heal the wound or remove a dead cell. Thus, the authors speculate that the lack of lamellipodia formation is the trigger for actomyosin cable contraction. Next, the authors examined repair dynamics in larger wounds. As observed in small multicellular wounds, lamellipodia and multicellular actin cables form at the wound edge, while actin cable contraction was not seen until a large gap was filled with lamellipodia through collective cell migration. During collective cell migration, cells adjacent to the wound exhibit enriched actin on the side of the wound. This actin enrichment spreads to ~10 cells by 60 min after wounding. This actin enrichment in submarginal cells is not observed in massive dead cells. Thus, diffusion signals from dead or damaged cells do not likely transfer information to neighboring cells.
This manuscript provides a description of what happens at different scales of wound healing in Clytia - which has several interesting features. However, the presented data do not fully support the authors' statements, especially regarding the proposed universal

mechanisms in epithelial wound repair.

Major comments:

(1) The authors describe different mechanisms that are required to close wounds in Clytia. Actomyosin ring contraction, not the lamellipodia extension, is required for micro wounds and small wounds with basement membrane damage. Both lamellipodia extension and actomyosin ring contraction are required for small wound closure without basement membrane damage. Collective cell migration is required for larger wound closure. These results suggest that multiple mechanisms are used to repair wounds of different sizes in Clytia. It is hard to understand how these different mechanisms are providing a single set of rules or single strategy for wound repair or what that means in terms of the repair process.

2-1 response:Thank you for this comment. It seems that the language we used did not successfully communicate our main take-home message, which is that there is a consistent decision tree that is applied to all wounds at all stages of healing in Clytia, and that determines the healing mechanism chosen in any given context.

To fix this and address this concern (and some of the concerns listed below) we have changed the title, abstract and text. Instead of using the terms "single strategy" or "set of rules", we now explain that we are defining a unified model and a coherent decision tree that determines the choice of wound healing mechanism for all wound types. We have also added a flow diagram (Figure 7B) to more completely explain our interpretations of our data. Some examples of text changes are below. We agree that this is an improvement and more accurately represents our data.

Title: The basement membrane determines the choice of wound healing mechanism across wound scales in the basal eucaryote Clytia hemiphaerica

Abstract: ..... One or more mechanisms are involved in the healing of a wound, yet the choice, sequence, and coordination of these processes are poorly understood. Moreover, different mechanisms dominate in different tissues, organisms, wound types and wound sizes, further complicating our understanding of how cells select among healing mechanisms. In this study we analyze wound healing across wound types and spatial scales within a single organism, the basal eukaryote *Clytia hemisphaerica* (Clytia), to establish a unified model for mechanism selection.

Significance Statement: - - Epithelial wound healing is crucial for the survival of multicellular organisms, yet the fundamental question of how cells select the best repair

strategies and coordinate these mechanisms in diverse wound types and sizes remains unclear.

- Using the transparent medusa of Clytia, we define a decision tree that determines how wound healing mechanisms are orchestrated in all wound types, and identify the availability of the basement membrane as a key regulator in the choice of mechanism.

Introduction: .... . Diverse mechanisms have been described for wound closure, but why one mechanism dominates over others in any given situation is unknown. In addition, in many cases more that one mechanism appears to be involved in closing a single wound[2], but how the mechanisms are coordinated to accomplish healing remains an unresolved question. ..... Comparison of actin dynamics and structures during healing in micro-wounds, small, and large wounds confirms that wounds of all types and sizes follow a coherent decision tree to determine the healing mechanism, and that the availability of exposed basement membrane plays a critical role in mechanistic decisions..... This integrated model for healing of wounds at all scales in Clytia highlights the ubiquitous and overlapping roles of lamellipodia and actomyosin purse strings in wound closure and demonstrates that the availability of the BM plays a critical role in orchestrating the essential events in wound healing.

Discussion

1. A coherent decision tree determines the healing mechanisms for epithelial wounds across scales in Clytia

Our observations lead to a model and decision tree that, together, explain the choice of wound healing mechanism for all wound types and spatial scales in Clytia, with the availability of the BM as a key determinant (Figure 7A).

(2) The descriptions presented here for wound repair in Clytia do not reach the level of those already published for other wound repair models. Robust quantification is required to support the authors' statement. Many conclusions in this manuscript rely on n=1 images with assumptions and without robust quantification. The images provided are not compelling enough to support the authors' statement, especially regarding the fluorescent images.

For example, in Figure 3, the authors state that actin exhibits the persistence of punctate patterns around wound gaps with debris at 5min. However, although those patterns are observed at 2.5 minutes, they do not appear at 1.5 and 3.5 minutes. Additionally, debris inside the wound is visible at 3.5 and 5 minutes, but not at 30 seconds, 1.5 minutes, or 2.5 minutes. The wound size at 1.5 minutes is smaller than at 2.5 and 3.5 minutes. Why does

debris appear at later time points in the presence of blebbistatin, either because the wounds cannot close or because another mechanism, such as a membrane patch model, is involved in the repair process?

2-2 response A: In Figure 3, the fluorescent images are of different wounds in different animals, and are representative images at each time point, so wounds will be of different sizes depending on the starting size, and will differ in the amount of debris. While the fact that actin staining shown in fluorescent tissues is in different animals for each timepoint is stated in the legends and methods, to avoid confusion we are adding the following to the text:

(Note that DIC images follow the closure of specific wounds over time, while fluorescent images show wounds from different animals fixed at the indicated timepoints.)

Our DIC and FL images show that lamellipodia advance and close micro-wounds completely and actin rings will not form in the presence of blebbistatin. In Figure 3, 5 min this is seen for all wounds between cells and many wounds within cells (now marked with arrows for clarity).

We agree with the reviewer that the punctate pattern of actin seen around some blebbistatin treated wounds at 5 minutes is poorly explained. We nevertheless wanted to imclude these images for completeness. It is possible that this is an imaging artifact and the puncta are simply tips of advancing lamellipodia. Alternatively, they may be identical to the small actin puncta that appear in a "firework" pattern at the last stage of healing in untreated wounds (ie Figure 1 3.5min, now noted in the Figure 1 legend). We have altered the legend to Figure 3 to state: In all wounds between cells and most wounds within cells (arrows, 5 min) lamellipdodia completely cover the wound gap by 5 minutes, although some wounds within cells close more slowly." This is accurate because in our timelapse videos we never observe micro-wounds that fail to close, with or without debris. We have further altered the text describing the Blebbistain results to make no assumptions about the actin puncta but only to state: " Occassionally, punctate actin is seen surrounding wounds with debris at time points when other wound gaps appear completely covered with lamellipodia (Figure 3A, 5 min); this may reflect the difficulty in healing such wounds without contraction to extrude debris. "

In Figure 1, the wound size at 1.5 minutes is also smaller than at 2.5 minutes.

2-2 response B: As above, in the fluorescent images we are not watching a single wound heal over time. Instead, we are fixing wouded animals at various time points post wounding. In each case we have tried to choose representative images that show examples of all the phenomena that we describe. However, since not all wounds start out

the same size, wounds from different animals may appear different at each time point. In contrast, the DIC images and videos show the progression of a single wound and its closure with and without debris (Figure 1C,D), and with blebbistatin in Figure 3B.

In Figure 1 we show the closure of wounds with and without debris using DIC images. The Fl images show examples of the actin structures that are found in a field of microwounds at various time points. In both the 2.5min and 3.5 min timepoints in Figure 1, we can see wounds that have completely closed and those with debris that appear to close more slowly. We have added a red arrow to the 2.5 min panel to highlight a closed wound.

How often are wounds with debris observed in different conditions, including drug treatment, and at different time points?

2-2 response C: The prevalence of wounds with internal debris was hard to predict across experiments, and we cannot explain why we saw it in some cases more than others. Nevertheless, we imaged extensive numbers of wounded animals with DIC and FL to be sure to capture all the possible types of wounds.

In Figure 4, the authors state that actomyosin contraction cannot fully close small wounds, but can close very small wounds. However, it is difficult to assess whether actin cables normally form in the presence of drugs, but cannot contract, or whether changing cell volume simply causes wounds to contract or fill the space regardless of actomyosin cable formation.

2-2 response D: In Figure 4F and G, where lamellipodia formation is inhibited by drugs, it is formally possible that neighboring cells expand in volume to reduce the wound gap, although the mechanism for such a volume increase within the short time frame of this experiment (<5min) is not immediately obvious. We don't believe this is the case because in the videos we can clearly see the pinching of the edges of marginal cells and the rounding of wound corners, all typical of cable contraction. We have amended the text to present this argument: "However, most small wounds show contraction (as evidenced by the decreasing area of the wound, the rounding of the wound shape and the pinching of the surrounding cells) but do not close even with long observation ((Figure 4F,G; Video 10,11)."

In Figure 6, the authors use dead cells to address whether a damage-induced diffusible signal is involved in the flow of information from marginal to submarginal cells. However, there is no citation showing that dead cells release the same damage-induced diffusible molecules when organisms are damaged to trigger wound healing processes. In addition,

no phalloidin staining is shown. Additional data, higher quality images, and robust quantification are needed to support the stated conclusions.

2-2 response E: We agree that killing cells by excitation of the CellMask fluorescent tag (likely leading to lethal ROS production, but we did not test this) is quite different than damaging cells by wounding, although we know of no study that has specifically addressed this. We have amended the text to reflect this caveat: Results section: "...although we cannot rule out the possibility that wounded cells produce different diffusible signals than cells killed with by phytotoxic dye." Discussion: "Nevertheless, it cannot be ruled out that different factors are released during wounding than in response to CellMask toxicity. "

Phalloidin staining of CellMask wounds is shown in Figure 6E but it was incorrectly referenced in the text – this has been fixed.

In regards to the more general comment about robust quantitation, it is certainly true that the data presented throughout this study is highly qualitative. In most cases, our data demonstrate the consistent presence or absence of various actin structures or cell behaviors. In all cases we are, by necessity, showing images and videos that are representative of many images and videos collected from many animals and wounds (in no cases is n=1). We have added a phrase to each legend stating that images are representative and stating the number of experimental replicates in each case.

In response to the reviewers comment that more quantitative analyses are needed we reviewed our work to identify any quantitative claims. There are very few. The claim that micro-wounds without debris close more quickly than those with debris is quantitated in Supplemental Figure S1. The claim that lamellipodia in micro-wounds treated with blebbistatin persist longer than in untreated micro-wounds has been changed to more clearly indicate that open wound gaps persist at timepoints when all wounds are closed in untreated animals, and that lamellipodia are seen in these open gaps, removing any quantitative claim: " Indeed, large lamellipodia, often overlapping, are observed in wound gaps at time points when wounds are completely closed in untreated animals (Figure 3A; compare 1.5min and later timepoints to Figure 1). " The claim that actin localization to the fronts of submarginal cells in large wounds spreads outward from the wound edge, and that this spread is greatly decreased if dead cells adhere to the wound gap, is quantitated in Figure 6H.

(3) It is not clear to what degree the effects of different sizes and shapes of wounds are taken into account. Previous studies showed that repair mechanisms differ between small and large wounds (PMIDs: 24482116 and 35736340). Wounding methods in this manuscript result in a wide range of sizes and shapes at different scales. In particular, the

drugs used in this study affect the cytoskeleton, thereby altering cell stiffness. In Figure 2D, all cells look like they are dissociating from each other, and a cell with low LatA is almost split in half. However, DIC images in 2A and 2B do not look like that. In addition, the shape of the wound is a factor in determining whether lamellipodia crowding or contractile ring formation during epithelial gap repair (PMID: 26158873). Indeed, all wounds are longitudinal shapes in Figure 4, whereas the wound shape in Figure 5 is rounded.

2-3 Response A: The reviewer is correct that some of the data and models in this paper contradict previous published studies, or perhaps reveal a unique feature of the Clytia system. This adds to the significance of the work

We have very carefully described, in Clytia, the mechanisms that are deployed to close wounds of different size and shapes. We do not see completely different mechanisms of wound closure in wounds of different sizes but integration of various mechanisms deptending on the specifics of the wound, as defined in the model and decision tree in Figure 7. In contrast to the some earlier studies in other systems including the work of Ravasio et al (2015) PMID: 26158873 cited by the reviewer and other works reviewed in Begnaud et al (2017) PMID27131272 (but not PMIDs: 24482116 and 35736340 listed by the reviewer, as these studies focus on plasma membrane damage within a single cell), our results do not support the model that shape is a major factor in determining the mechanism of wound closure in Clytia.

Indeed, all wounds are longitudinal shapes in Figure 4, whereas the wound shape in Figure 5 is rounded.

2-3 Response B: The different shape and closure mechanism of the wounds shown in Figures 4 and 5 perhaps led to some confusion. In Figure 4, we show examples of small wounds, which are always irregular and diverse in shape but nevertheless heal by the same series of responses – lamellipodia extension until the lamellipodia meet, provided that they have a basement membrane to crawl on, followed by contraction of an actin cable that forms while the lamellipodia are extending. In contrast, the small wounds shown in Figure 5 have a damaged (A) or occluded (B) basement membrane. In these cases the lamellipodia cannot extend and the wounds are closing by actin cable "purse string" contraction, which will create a circular shape as it pulls the wound closed. Hopefully our new description of a consistent decision tree that determines the healing mechanism, together with our new flow diagram (Figure 7B) will better clarify this point. We have also compared figures 4 and 5 wound as follows: Multicellular actomyosin purse strings have been extensively described in the closure of small wounds[3], [4], [5], [6], [7], [8], [9], [10], [12]. The "purse string" nomenclature reflects that they usually are seen surrounding and closing a circular gap, reminiscent of the top of a purse. In Clytia, we previously reported

that lamellipodia rapidly retract when they encounter BM damage in a wound gap, and the wound subsequently closes through a purse string mechanism [34] (Figure 5A; Video 12). A similar response is seen when a dead cell occludes the BM; in this case an actomyosin cable surrounds the dead cell and contraction extrudes the damaged tissue (Figure 5B,C; Video 13). Notably, the contraction of a multicellular purse-string in these examples is essentially identical to actomyosin cable contraction during the final healing stage in small wounds (ie Figure 4A) except that contraction around a damaged BM area or dead cell typically appears circular, while contraction in wounds already closed by lamellipodia appears linear (Figure 4A vs Figure 5). Further, actomyosin cables are evident in all small wounds, even in the absence of BM damage or dead cells (Figure 4). Collectively, these findings indicate that multicellular actomyosin purse strings are not exclusive to circular wounds or those with BM damage or occlusion; rather, they are integral to the healing process in all small wounds in *Clytia*.

The reviewer is also correct in observing that Latrunculin, particularly at high concentrations, has a highly destructive effect on tissues and cells. Nevertheless we were able to consistently identify regions with intact fields of cells to analyze, including those shown in Figure 2. Despite the intensive damage, actin was able to localize to the wound periphery and purse strings were able to close the majority of wounds in the low, but not high, LatA treatment.

(4) The wounds generated are highly variable in the number and types of wounds. Do wounds within a cell heal with the same dynamics when it is attached to its neighboring cells compared to cases where many of the cells have been ripped apart from each other?

2-4 Response: In micro-wounds, the only circumstance where cells are ripped apart from their neighbors is with the LatA treatment. Figure 1B shows a particularly dramatic field or cells with microwounds, and although there are many wound between the cells the cells never completely separate.

(5) Diverse mechanisms proposed in different models need to be considered. Previous studies using different models and wounding methods have suggested that organisms have distinct mechanisms for responding to various sizes and types of wounds to heal efficiently. Even in similar contexts of wound healing, different mechanisms have been proposed previously. For example, while actomyosin ring translocation is highly conserved for cell wound closure, the mechanisms of translocation are not identical and include Myosin-dependent contraction in the Drosophila embryo model, actin treadmilling in the Xenopus oocyte model, and Arp2/3-dependent actin assembly in the C. elegans model.

2-5 Response A: We appreciate this comment, as it agrees with our own view that the literature is complex due to the many different models and wound types that are studied. As reviewer 1 points out, it is currently unclear if every system heals epithelial wounds in a unique way or if the different wound types studied obscures conserved strategies. We hope to contribute to the literature by defining the decision tree that determines the wound healing mechanism for all wound types in a singe organism (Clytia), identifying unappreciated nodes such as the availability of the basement membrane, as well as new regulatory steps that may be conserved such as the triggering of a previously formed actin cable.

The author's proposal that Clytia serves as a universal model for all wound repair is not supported by their data and is an overstatement.

2-5 Response B: Our model offers a quite new way of thinking about healing mechanisms, and the factors that drive the choice between them. We realize that this particular model may not hold true for all organisms, as discussed in the final section of the discussion. However, by creating this model we hope it might serve as a paradigm for similar studies in more well-studied systems, and may lead to hypotheses that could be tested directly in vertebrate systems. We agree that "universal model" was a poor choice of words and we have removed that and similar terms throughout the text. Our revised last paragraph perhaps better reflects these ideas:

The reports of co-existing lamellipodia and actin cables in vertebrate tissues [11], [45] and the well-documented participation of both lamellipodia and purse strings in wound healing in vertebrate epithelia cells [2], [4], [5], [6], [7], [11], [44] indicates that evolutionarily distant organisms face the same regulatory challenges in selecting between lamellipodial and purse string mechanisms of wound closure. However, there are many examples that implicate very different decision nodes in other systems. For example, wounds in mouse early embryos heal through purse string closure but at stage E15 transition to healing with lamellipodia[52], suggesting a changing developmental context. In coelomycetes of sea urchin, micro-wounds that go all the way through the lamellipodia and seal, similar to the microwounds we describe in Clytia, heal without forming an actomyosin cable[53], perhaps providing an example of a tissue-specific wound-healing strategy. It is also reasonable to speculate that more complex regulatory mechanisms for sensing and responding to different types and sizes of wounds may have evolved over time. Despite these caveats, that Clytia model presented here allows us to predict how cellular behaviors are orchestrated in many wound healing contexts. This should lead to testable hypothesis in vertebrate and other systems, which in turn may reveal unsuspected conserved strategies and regulatory steps in optimizing epithelial wound healing.

Dear Dr. Malamy,

Your manuscript, "A single strategy describes healing of all epithelial wound types in the basal eukaryote Clytia hemisphaerica" has now been evaluated by two experts. As you will see, they differ in their opinion as to the suitability of this study for publication in MBoC, with reviewer 1 having only one minor concern and reviewer 2 having several major concerns. However, a close inspection of the comments of reviewer 2 as well as your manuscript leads me to believe that nearly all of reviewer 2's comments can be addressed by textual revisions. Accordingly, we would be willing to consider a revised manuscript that addresses each of the points below.

1. In reference to the various processes you have reported as being involved in wound repair, Reviewer 2 writes "It is hard to understand how these different mechanisms are providing a single set of rules or single strategy for wound repair or what that means in terms of the repair process".

The issue here seems to be one of wording. Presumably, you do not mean that there is no difference between multicellular purse strings, lamellipodia and collective cell migration and yet this is what is implied by your title and the language you use at several points in this manuscript. Moreover, you also point out that the healing response differs in the presence and absence of the basement membrane which surely implies that there is more than one "rule" or "strategy". We therefore ask that you rework the title and the relevant places in the text to avoid giving the impression that only a single mechanism, strategy or rule is in play. Possibilities include referring to lamellipodia and purse strings being used in a consistent fashion across different wound scales. You may also want to incorporate the basement membrane into the title as that will increase the interest in your study, but that is up to you.

Thank you for this comment. We have changed the title as suggested and have altered the language throughout the paper. We no longer use terms "universal", "rules" or "strategies" as these seemed to generate confusion. Instead we discuss a model/decision tree that determines the choice of wound healing mechanism across wound scales. More details are above in response to reviewer 2 (2-1 Response). Changes to the title and text also highlight the key finding that basement membrane availability is a critical regulatory of mechanism choice. In the decision tree added as Figure 7B, the central placement of the "basement membrane availability" node will further emphasize this result.

2. Reviewer 2 points out that it is not clear how many experiments your findings are based on and that there is insufficient quantification. This concern can be addressed by including quantifications as well as statistical analyses for any quantitative claims. In some cases where claims are qualitative or difficult to quantify, it may be sufficient to state that results shown are representative of X number of experiments; failing this, such claims should be dropped.

As stated in response Reviewer 2 comment 2 (2-2 Response E), we believe that we have provided data to support all of our quantitative claims, except for one case which we have removed. For qualitative data we now describe experimental replicates in the legends and indicated that images are representative.

3. Reviewer 2 raises the question of the potential impact of wound size and shape on the interpretation of the results. This concern can be addressed by acknowledging that the wounds vary in terms of size and shape and by discussing whether these differences have any apparent impact on the results.

We believe we have addressed this concern in 2-3 Response A and B.

4. Similar to point 3, Reviewer 2 notes that there are varying numbers of wounds produced in the experiments used to generate "microwounds" (ie full-thickness wounds of individual cells) and wants to know if the healing process differs as a function of nearness to other wounds. This concern can also be addressed by acknowledging that the number of wounds varies and by discussing whether this variation impacts the reported results.

See 2-4 Response above.

5. Reviewer 2 points out that there is good evidence that there is fundamental variation in how healing occurs in different model systems. For that reason, Reviewer 2 feels that claims of universality based on the findings presented here are an overstatement. This concern can be addressed by toning down or removing claims of universality (see also below). We did not intend to imply that the mechanisms demonstrated in Clytia would necessarily be the same in all other organisms, and have corrected the language throughout to remove this impression, and re-written the final paragraph of the discussion. See also 2-5 Response A and B.

6. My impression after reading your study is that you could strengthen its impact with revisions that increase clarity and reduce the potential for misunderstandings about

previous work. Some of this will be accomplished by addressing point 1 above. For example, it took several readings and repeated viewing of Figure 7 before your main point sank in. Additionally, it would be to your advantage to focus on what is truly new here. For example, the statement "The co-existence of lamellipodia and actomyosin structures surrounding wounds is counter to the general assumption that cells will have one or the other of these actin-based structures driving wound healing" might lead readers to believe that you consider this one of the novel findings of your study. However, the dynamic coexistence of purse strings and lamellipodia in the same wound was reported for healing of cultured human cell monolayers more than 30 years ago (pmid 8486737) and for Drosophila embryos more than 20 years ago (pmid 12402048). Thus, setting up lamellipodia and purse strings as "either/or" seem like a strawman to those familiar with the wound healing literature. Moreover, a little later in the Discussion, it is made clear that this really is a strawman when the reader is told " â€¦ the implication of cooperation between lamellipodia and purse strings in wound healing in vertebrate epithelia cells [3], [4], [5], [6], [7], [11], [50] suggests that the universal strategy for epithelial wound healing uncovered in Clytia may be widely conserved". Fortunately, this problem could be easily fixed by replacing the "either or" construct with a simple statement to the effect that while wounds have long been known to use purse strings and lamellipodia simultaneously to drive healing, the temporal deployment pattern of these mechanisms across different scales and conditions is poorly understood.

We have taken all of these excellent criticisms to heart and done some rewriting of the text. We now immediately acknowledge, in the abstract and introduction. that data from many systems shows that multiple mechanisms cooperate in healing wounds, while pointing out that the question of how the mechanisms are orchestrated and how they interact with eachother is a major knowledge gap. We have also changed wording to emphasize that our results present a model for how the healing mechanism(s) are selected for any given wound in Clytia. See our 2-1 Response for some examples of this. Furthermore, we have updated Figure 7A for clarity and added a decision tree as Figure 7B to better communicate our ideas.

Another area of the text that seems likely to be misunderstood is the difference between the microwounds in this study and single cell wounds in most other studies. The microwounds in this study are apparently not holes in the cell surface on one side of the cell, but full thickness wounds such that the hole is surrounded on all sides by intact plasma membranes. Because of the growing popularity of single cell repair as an object of study, this point should probably be emphasized in the discussion when microwounds are first discussed.

We refer to our wounds as "micro-wounds" rather than "single-cell wounds" to make this distinction, and define them explicity in the Intro and Results sections already. We have now added a note to the discussion as follows: "The parallels between actin rings and multicellular actin cables in various types of wound healing have been recognized before. For example, other researchers have pointed out that single-cell wounds in the plasma membrane heal through contraction of an actin ring, and noted the similarity to actin purse-strings[40], [41]. (Note that the single-cell wounds referred to in these studies are holes in the cell surface, in contrast to micro-wounds described here in Clytia that pass all the way through all cell membranes and create a hole that exposes the BM underlying the epithelial monolayer). "

Additionally, a key report in which similar full thickness wounds were studied is missing from the Discussion. That is, Henson et al. (2002; pmid 11907278) studied the healing of full thickness wounds in sea urchin coelomocytes. In that study the authors reported that such holes heal solely by lamellipodial extension with no evidence for purse strings. Why the results of that study differ from those of the current study is not clear, but this point needs to be addressed. Moreover, the results of that study (taken together with the comments of Reviewer 2) underscore the potential danger of overstating the generality of the findings of this study.

We cannot explain the discrepancy beyond noting that this is a different type of wound in a different organism. Our revised final paragraph of the discussion acknowleges the likelihood of diverse mechansims and strategies among organisms using this study as an example, but nevertheless offers the possibility that our comprehensive model for selecting wound healing mechanisms in a wide variety of contexts in Clytia can serve as a baseline for future studies in diverse systems.

We sincerely feel that the feedback and resulting changes have made our manuscriot considerably stronger. With these changes we hope our paper is now acceptable for publication.

Sincerely;

RE: Manuscript #E26-02-0094R
TITLE: "The basement membrane determines the choice of wound healing mechanism across wound scales in the basal eukaryote Clytia hemiphaerica"

Dear Jocelyn,

Many thanks for quickly and thoroughly addressing all of the points raised by the reviewers and by the handling editor. I am pleased to tell you that your manuscript is now acceptable for publication by MBoC.

Thank you for submitting this interesting and important study to MBoC!

Bill Bement
Monitoring Editor
Molecular Biology of the Cell

Dear Dr. Malamy:

Congratulations on the acceptance of your manuscript! Thank you for publishing your work in Molecular Biology of the Cell (MBoC).

Within 10 days, an unedited PDF of your manuscript will be published on MBoC in Press, an early-release journal version. The date your manuscript appears on this site is the official publication date.

Your copyedited and typeset manuscript will be scheduled for publication in the next available issue of MBoC. Once your paper is ready for review, our production team will notify you. In the summer of 2024, our production provider will introduce an online proofing solution, which will streamline the process of checking and validating author changes. This will result in improved typesetting quality, enhanced digital content, and a faster overall process.

We invite you to submit images related to your accepted manuscript to be considered for the journal cover.
Cover images must contain color, TIF Format
Size image at 7.4" wide x 5.85" deep
RGB and CMYK color (please submit two separate files, one in each format)
300 dpi resolution

Please also send a 1-paragraph description of the image that a general audience could understand. Include with the caption the name and institution of the individual(s) the image should be attributed to. Please provide this description in a Word file. You can send the file via email or by your favorite file transfer service to mboc@ascb.org.

Authors of Articles and Brief Communications are also encouraged to create a short video abstract to accompany their article when it is published. These video abstracts, known as Science Sketches, are up to 2 minutes long and will be published on YouTube and then embedded in the article abstract. Science Sketch Editors on the MBoC Editorial Board will guide you as you prepare your video. Information about how to prepare and submit a video abstract is available at www.molbiolcell.org/science-sketches. Please contact mboc@ascb.org if you are interested in creating a Science Sketch.

We look forward to publishing your paper in MBoC.
Sign up for MBoC's new content alerts here https://tinyurl.com/3bcsyk3e

Sincerely,

Eric Baker
Journal Production Manager
MBoC Editorial Office
mbc@ascb.org
